# A Single‐Amino‐Acid Ligand for LAT1: A Minimalist and Modular Platform for Lysosome‐Targeted Degradation of Membrane Proteins

**DOI:** 10.1002/advs.76862

**Published:** 2026-08-03

**Authors:** Liquan Zhu, Ke Liu, Haotian Liu, Chaoqi He, Xiaozhen Liu, Xin Zeng, Misha Mao, Yuxiao Mu, Ying Li, Qinghui Zheng, Hongchao Tang, Da Qian, Xuli Meng

**Affiliations:** ^1^ Department of Breast Surgery, General Surgery, Cancer Center Zhejiang Provincial People's Hospital Affiliated People's Hospital Hangzhou Medical College Hangzhou Zhejiang China; ^2^ Key Laboratory for Diagnosis and Treatment of Upper Limb Edema and Stasis of Breast Cancer Hangzhou Zhejiang China; ^3^ Central Laboratory, Department of Burn and Plastic Surgery‐Hand Surgery Changshu Hospital Affiliated to Soochow University Changshu No.1 People's Hospital Changshu Jiangsu China

**Keywords:** amino acid ligand, LAT1, lysosome‐targeting chimera, membrane protein degradation, modular degradation platform, targeted protein degradation

## Abstract

Targeted degradation of membrane proteins via the lysosomal pathway holds great therapeutic promise, yet existing platforms rely on bulky ligands such as glycopolymers, antibodies, or protein nanocages, which complicate synthesis and limit tissue penetration. Here, we repurpose L‑type amino acid transporter 1 (LAT1)—a nutrient transporter overexpressed in diverse cancers—as a lysosomal targeting receptor. Taking advantage of LAT1‘s natural substrate preference, we design a minimalist chemical handle: a single phenylalanine derivative that serves as a high‑affinity LAT1 ligand. This ligand is conjugated via bioorthogonal chemistry to various warheads (antibodies or small molecules) to generate modular degraders termed LAT1‐mediated lysosome‐targeting chimeras (LA‑LYTAC). These chimeras efficiently internalize and route oncogenic membrane proteins—including PD‑L1, EGFR, and integrins—to lysosomes for degradation. The platform operates through a LAT1‑dependent, lysosomal mechanism and suppresses downstream signaling pathways. In a syngeneic mouse model of triple‑negative breast cancer, a PD‑L1‑targeting LA‑LYTAC reduces tumor growth by more than 60%, enhances CD8^+^ T cell infiltration, and shows no overt toxicity. This work establishes a synthetically accessible, truly modular, and tumor‑selective degradation platform driven by a single amino acid, offering a streamlined alternative to existing lysosome‑targeting technologies.

## Graphical abstract

LA‐LYTAC repurposes the cancer‐enriched amino acid transporter LAT1 as a lysosomal trafficking receptor for targeted membrane‐protein degradation. A single phenylalanine‐derived ligand, modularly linked to antibodies or small‐molecule binders, recruits PD‐L1, EGFR, or integrins to LAT1, triggering transporter‐mediated internalization, lysosomal degradation, LAT1 recycling, and selective antitumor responses.

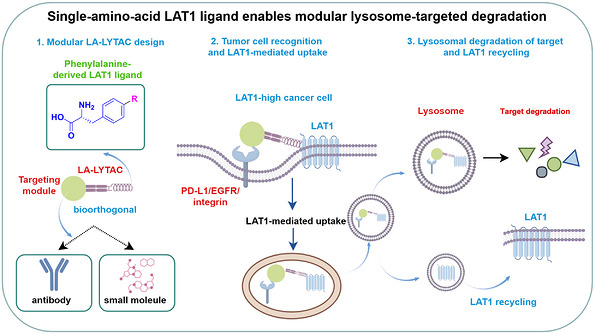

## 1 Introduction

Targeted protein degradation (TPD) has emerged as a powerful modality for eliminating pathogenic proteins by hijacking the cell's own degradation machinery [1]. Among the two major proteolytic routes—the ubiquitin–proteasome system (UPS) and the lysosomal system—the latter is uniquely capable of processing membrane proteins, extracellular aggregates, and even entire organelles via endocytosis and autophagy [2, 3, 4]. This capability has spurred the development of lysosome‑targeting chimeras (LYTACs) [5], antibody‑based PROTACs (AbTACs) [6, 7], and other bifunctional degraders that recruit a protein of interest (POI) to a cell‑surface recycling receptor [8], thereby redirecting the POI to lysosomes for degradation.

Most of these systems rely on antibodies or their fragments, owing to their modular design, high target specificity, and broad applicability [9]. Although powerful, antibody‑based degraders face two interconnected challenges. First, chemical conjugation or genetic fusion of the antibody to a receptor‑binding moiety often compromises its native binding affinity and complicates manufacturing [10, 11]. Second, the large hydrodynamic size of antibodies limits their penetration into solid tumors, resulting in suboptimal efficacy [12]. Small‑molecule degrader systems, such as molecular degraders of extracellular proteins (MoDEs) and integrin‑facilitated lysosomal degradation (IFLD) [13, 14, 15], offer improved tissue permeability. However, their small size leads to rapid renal clearance and necessitates frequent dosing. Moreover, their target scope is inherently restricted because only a minority of membrane proteins have known small‑molecule ligands with sufficient affinity and specificity that can be engineered into degraders [16].

To overcome these limitations, we sought a recycling receptor that combines tumor‑selective overexpression with the ability to be engaged by simple, synthetically accessible ligands—ideally, a small molecule that is both a minimal chemical handle and a high‑affinity binder. We identified L‑type amino acid transporter 1 (LAT1, *SLC7A5*) as an ideal candidate [17]. LAT1 is a heterodimeric transporter (with CD98) that mediates the uptake of large neutral amino acids such as leucine and phenylalanine [18]. It is markedly upregulated across diverse malignancies, including breast, lung, glioma, and pancreatic cancers, where its expression correlates with tumor growth, invasiveness, and poor prognosis [19, 20]. Importantly, LAT1 undergoes constitutive endocytosis and recycling; ligand engagement promotes internalization, with a fraction of the transporter returning to the plasma membrane while the remaining cargo is delivered to lysosomes. This trafficking behavior mirrors that of established lysosomal targeting receptors such as CL‑M6PR and ASGPR [21, 22], yet LAT1 offers a decisive advantage: its natural substrates are amino acids. Consequently, a single synthetic amino acid derivative can serve as a high‑affinity LAT1 ligand—a minimal, readily derivatizable chemical motif that avoids the synthetic complexity of glycopolymers or multivalent glycans used in conventional LYTACs.

The recent surge of lysosome‐targeting degraders has yielded several conceptually distinct platforms. Notably, FRTACs harness folate receptor α (FRα) as a novel lysosome‐trafficking receptor and employ a polyvalent crosslinking strategy—multiple folate molecules conjugated to an antibody—to achieve high‐avidity binding and subnanomolar degradation potency [23, 24]. CPPTACs take an orthogonal approach by exploiting the intrinsic endosomal entrapment of cell‐penetrating peptides (CPPs), thereby driving degradation in a manner independent of specific lysosomal trafficking receptors [25]. More recently, HFn‐LYTACs utilize human heavy chain ferritin (HFn) as a protein cage scaffold that exploits transferrin receptor 1 (TfR1)‐mediated endocytosis, combining nanoparticle size effects with multivalent ligand display [26].

While these platforms represent important conceptual advances, each carries inherent chemical and biological complexities. FRTACs require the synthesis of polyvalent folate‐antibody conjugates, which demands precise control of stoichiometry and introduces significant synthetic overhead. The polyvalent design, though effective for avidity enhancement, complicates manufacturing and may contribute to immunogenicity concerns. CPPTACs, while bypassing receptor dependence, rely on CPPs—cationic peptides of 8–30 amino acids—whose net positive charge can lead to non‐specific membrane interactions and potential off‐target effects. HFn‐LYTACs, built upon a 24‐subunit protein nanocage, present the largest molecular architecture among these platforms, which may pose challenges for tissue penetration and large‐scale production [23, 24, 25, 26].

In contrast, our LAT1‐based platform offers a fundamentally simpler chemical design. Rather than employing polyvalent scaffolds, cationic peptides, or protein nanocages, we use a single phenylalanine derivative—a molecule that mirrors LAT1‘s natural substrate—as the exclusive lysosomal targeting handle [27, 28]. This “minimalist” approach reduces the degrader to its essential chemical components, eliminating the need for complex multivalent conjugation, lengthy peptide synthesis, or recombinant protein expression. The synthetic accessibility, minimal molecular footprint, and low immunogenic potential of a single amino acid motif set our platform apart from these alternative strategies, positioning it as an attractive tool for chemical biology and potential therapeutic translation.

Here, we repurpose LAT1 from a nutrient transporter into a lysosomal targeting receptor for TPD, and we introduce a modular platform termed LA‑LYTAC. Using a compact amino acid derivative as the universal LAT1‑binding handle [29], we conjugate it via bioorthogonal chemistry to various targeting modules—either antibodies or small‑molecule inhibitors—directed against oncogenic membrane proteins such as PD‑L1, the epidermal growth factor receptor (EGFR), and integrins. The resulting chimeras drive rapid internalization and lysosomal degradation of the target proteins, while LAT1 recycles to the membrane for sustained activity. This “minimalist” design not only expands the repertoire of lysosomal trafficking receptors but also provides a truly plug‑and‑play platform that is synthetically simple, modular, and readily adaptable to a wide range of extracellular and membrane‑bound targets. We demonstrate that LA‑LYTAC achieve near‑complete degradation of PD‑L1 and EGFR in cancer cells, and we validate their antitumor efficacy in a syngeneic mouse model. To our knowledge, this work represents the first example of using a single‑amino‑acid ligand to drive lysosomal degradation via a metabolically‑upregulated transporter, opening a new avenue for the design of minimalistic, tumor‑selective protein degraders.

## 2 Results and Discussion

### 2.1 LA^Biotin^ Mediates Internalization and Lysosomal Degradation of Soluble Extracellular Proteins

**FIGURE 1:**
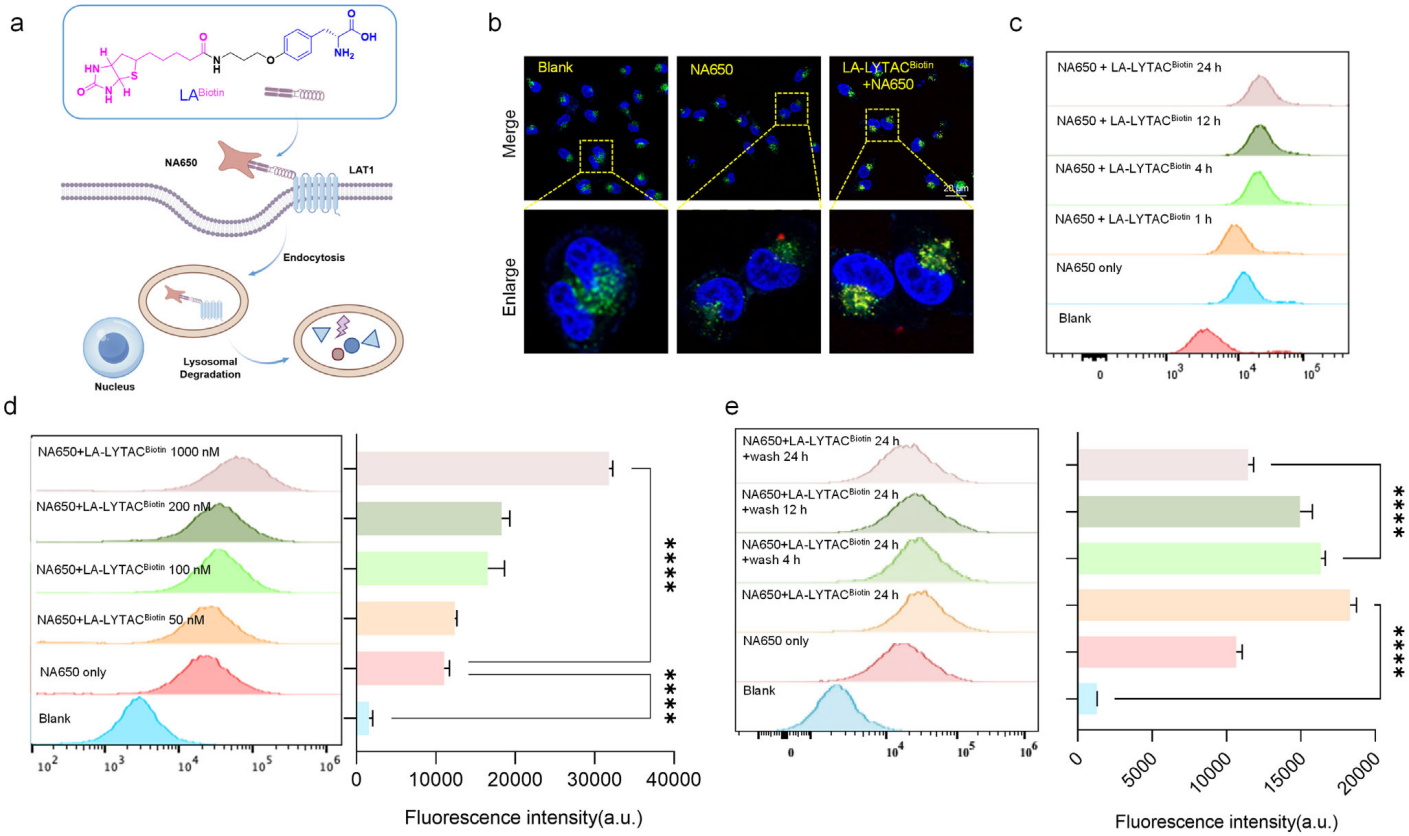
LA‐LYTAC^Biotin^‐mediated internalization and degradation of NA650. (a) Schematic diagram of the degradation of soluble extracellular proteins mediated by LA‐LYTAC. (b) CLSM images of HeLa cells incubated with 50 nM NA650 and LA‐LYTAC^Biotin^ at 37°C for 24 h. Red: NA650; Green: LysoTracker; Blue: Hoechst; Inset: DIC; Merge: overlay of different fluorescence channels. Scale bar = 20 µm. (c) HeLa cells were treated with 50 nM NA650 and 1000 nM LA‐LYTAC^Biotin^ for different durations (24, 12, 4, and 1 h), and the internalization of NA650 was analyzed by flow cytometry. (n = 3 per group). (d) HeLa cells were treated with 50 nM NA650 and different concentrations of LA‐LYTAC^Biotin^ (50, 100, 200, and 1000 nM) for 24 h, and the internalization of NA650 was analyzed by flow cytometry (*n* = 3 per group). (e) Flow cytometry analysis of NA650 uptake in HeLa cells assisted by LA‐LYTAC^Biotin^. Cells were treated with 1000 nM LA‐LYTAC^Biotin^ and 50 nM NA650 for 24 h, and then cultured in fresh medium for the specified time points (24, 12, and 4 h). (*n* = 3 per group).

To evaluate whether LAT1 can serve as a lysosomal targeting receptor for extracellular proteins, we first needed a suitable LAT1‑binding ligand. LAT1 naturally transports large neutral amino acids such as phenylalanine and leucine with high affinity and exhibits pronounced substrate promiscuity, tolerating various aromatic and aliphatic modifications at the amino acid side chain or termini. Based on this, we selected a phenylalanine derivative as the minimal LAT1‑targeting handle: it retains high binding affinity for LAT1, is synthetically accessible, and can be readily conjugated to other moieties via bioorthogonal chemistry. Using this handle, we designed a modular degrader platform termed LA‑LYTAC (LAT1‑mediated lysosome‑targeting chimera). As an initial proof‑of‑concept, we conjugated the phenylalanine‑based LAT1 ligand to biotin, generating LA^Biotin^ Figure S14, which captures a model extracellular protein (NA650) through streptavidin bridging (Figure 1a).

We first visualized the internalization and subcellular trafficking of NA650 by confocal laser scanning microscopy (CLSM). HeLa cells were incubated with NA650 (50 nM) in the presence or absence of LA^Biotin^ (50 nM) for 24 h. In the absence of LA^Biotin^, NA650 fluorescence remained predominantly on the plasma membrane, indicating minimal spontaneous uptake. In contrast, co‑incubation with LA^Biotin^ led to robust intracellular accumulation of NA650, which extensively co‑localized with LysoTracker, a marker of acidic lysosomal compartments (Figure 1b, Figure S1B). This co‑localization suggests that the internalized NA650 is efficiently delivered to lysosomes for degradation. Similar results were obtained in MDA‑MB‑231 cells, a triple‑negative breast cancer cell line with high LAT1 expression, where LA^Biotin^ also mediated NA650 internalization and lysosomal co‑localization (Figure S1A). A time‑course CLSM experiment in HeLa cells (4, 12, and 24 h) visually corroborated the gradual accumulation of NA650 in lysosomal compartments over time (Figure S1F).

We next examined the time‑dependent internalization by flow cytometry. HeLa cells were treated with NA650 (50 nM) and LA^Biotin^ (1000 nM) for 1, 4, 12, and 24 h. NA650 uptake increased progressively over time, reaching a plateau by 12–24 h (Figure 1c, Figure S1E). The concentration dependence was assessed by treating HeLa cells with NA650 (50 nM) and increasing concentrations of LA^Biotin^ (50–1000 nm) for 24 h. Flow cytometry revealed a dose‑dependent increase in NA650 internalization, with maximal uptake at 1000 nM (Figure 1d). Even at the lowest tested concentration (50 nm), LA^Biotin^ significantly enhanced NA650 uptake compared to the control, highlighting the high efficiency of LAT1‑mediated endocytosis. Similar dose‑dependent internalization was observed in MDA‑MB‑231 cells treated with NA650 and increasing concentrations of LA^Biotin^ (50–1000 nm) for 24 h (Figure S1C).

To assess the degradation kinetics, we performed a pulse‑chase experiment. HeLa cells were co‑treated with NA650 (50 nM) and LA^Biotin^ (1000 nM) for 24 h, then transferred to fresh medium without either reagent. Cells were harvested at 4, 12, and 24 h post‑chase and analyzed by flow cytometry. NA650 fluorescence intensity declined progressively, with approximately 50% reduction after 24 h (Figure 1e). This sustained clearance is consistent with a mechanism in which the target protein is delivered to lysosomes for proteolysis, while LAT1 recycles to the plasma membrane for subsequent rounds of internalization. A parallel pulse‑chase experiment in MDA‑MB‑231 cells showed a comparable time‑dependent decline in NA650 fluorescence, with approximately 45% reduction after 24 h (Figure S1D).

Collectively, these data demonstrate that LA^Biotin^, built upon a phenylalanine‑based LAT1 ligand, effectively mediates the internalization of a soluble extracellular protein via LAT1‑dependent endocytosis and directs it to lysosomes for degradation. The dose‑dependent uptake and sustained clearance kinetics, validated across two LAT1‑high cell lines (HeLa and MDA‑MB‑231), establish LAT1 as a functional lysosomal targeting receptor and provide a solid foundation for the LA‑LYTAC platform in targeted degradation of extracellular pathogenic proteins.

### 2.2 LA‐LYTAC^Ab^ Mediates Efficient Degradation of PD‐L1 in Triple‐Negative Breast Cancer Cells

**FIGURE 2:**
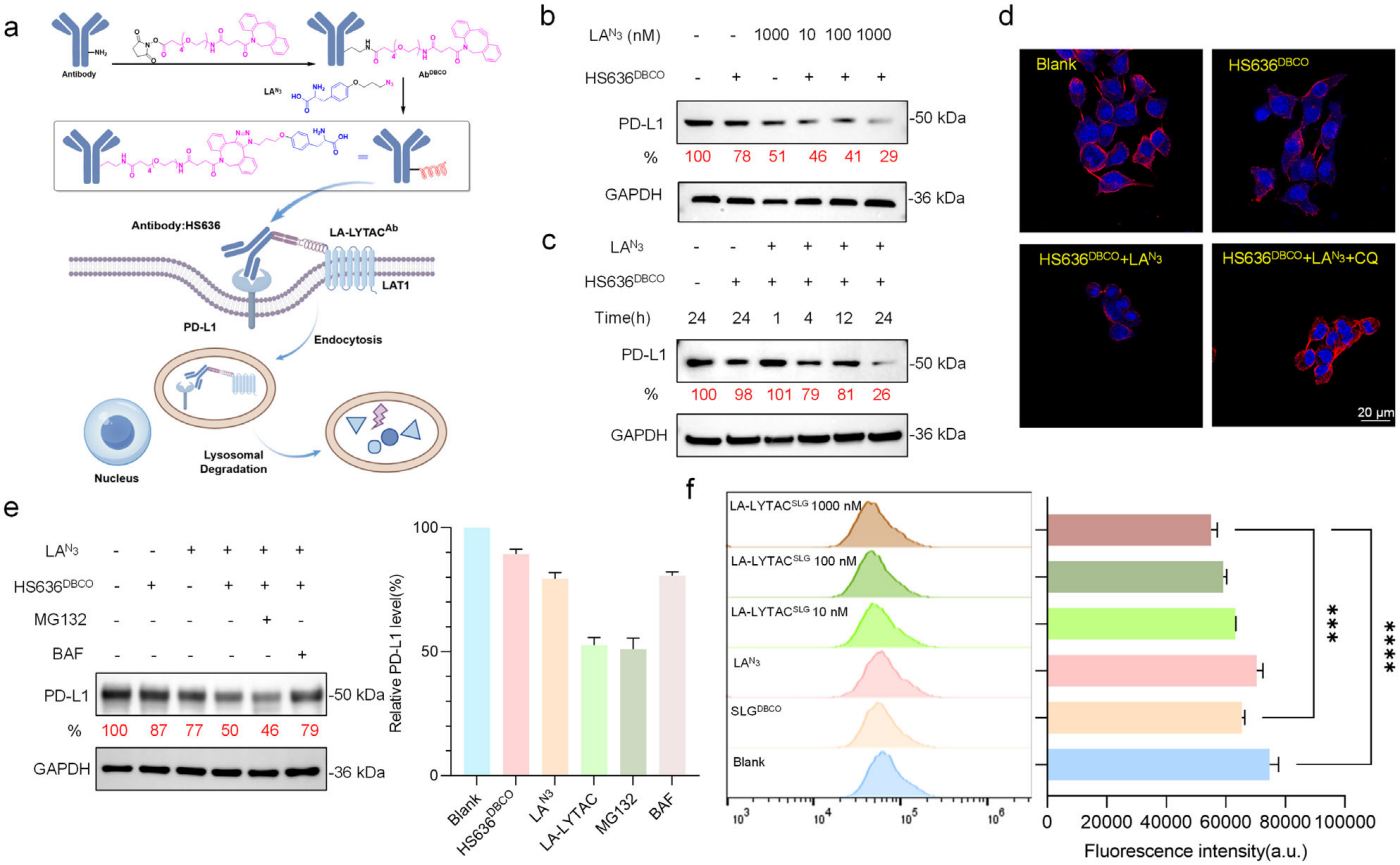
LA‐LYTAC^Ab^ promotes the degradation of PD‐L1 in the 4T1 cell line. (a) Schematic diagram of PD‐L1 degradation mediated by LA‐LYTAC^Ab^. (b, c) Western blot analysis of PD‐L1 degradation in 4T1 cells by LA‐LYTAC^Ab^, (b) cells treated with 10 nM HS636^DBCO^ and different concentrations LA^N3^ for 24 h, (c) cells treated with 10 nM HS636^DBCO^ and 1000 nM LA^N3^ at different time points. (d) Confocal microscopy images of PD‐L1 degradation in 4T1 cells after treatment with 10 nM HS636^DBCO^ and 1000 nM LA^N3^ for 24 h. Scale bar: 20 µm. (e) Western blot of PD‐L1 degradation in MDA‐MB‐231 cells after incubation with 10 nM HS636^DBCO^ and 1000 nM LA^N3^ with 1 µM MG132 or 1 µM bafilomycin (BAF) for 24 h. (f) Flow cytometry analysis of PD‐L1 degradation in 4T1 cells treated with different concentrations of LA‐LYTAC^SLG^, LA^N3^ and SLG^DBCO^.

Having validated the LA‑LYTAC platform for delivering soluble extracellular proteins to lysosomes, we next applied this strategy to degrade a clinically relevant oncogenic membrane protein. Programmed death‑ligand 1 (PD‑L1) is a key immune checkpoint molecule frequently overexpressed on the surface of triple‑negative breast cancer (TNBC) cells, where it mediates immune evasion [30, 31]. However, therapeutic anti‑PD‑L1 antibodies often exhibit poor internalization, limiting their ability to durably reduce cell‑surface PD‑L1 levels. We therefore designed LA‑LYTAC^Ab^ by conjugating the LAT1‑targeting small‑molecule ligand LA^N3^ (a phenylalanine derivative, Figures S15–S17) to the anti‑PD‑L1 antibody HS636 via bioorthogonal chemistry (HS636^DBCO^ + LA^N3^), enabling simultaneous engagement of PD‑L1 and LAT1 on the tumor cell surface (Figure 2a). To calculate the labeling ratio, Cy5‐N_3_, was used instead of LA^N3^, and the resulting antibody conjugate was analyzed by SDS‐PAGE/in‐gel fluorescence scanning.24 As a proof‐of‐principle experiment, Atezolizumab (ATZ)—a therapeutic antibody against PD‐L1, was chosen for the preparation of ATZ‐Cy5, resulting in an average labeling ratio of 5 (Figure S3A/B).

To assess PD‑L1 degradation, murine 4T1 TNBC cells were treated with a fixed concentration of HS636^DBCO^ (10 nM) in the presence of increasing concentrations of LA^N3^ (10, 100, and 1000 nm) for 24 h. Western blot analysis of whole‑cell lysates using an anti‑PD‑L1 antibody revealed that PD‑L1 protein levels decreased in a LA^N3^‑concentration‑dependent manner, with near‑complete degradation observed at 1000 nM LA^N3^ (Figure 2b). Neither HS636^DBCO^ nor LA^N3^ alone significantly affected PD‑L1 levels, confirming that the intact LA‑LYTAC^Ab^ chimera is required for degradation. Similar results were obtained in human MDA‑MB‑231 TNBC cells (Figure S2C). We next examined the temporal dynamics of cell‑surface PD‑L1 degradation. 4T1 cells were treated with HS636^DBCO^ (10 nM) and LA^N3^ (1000 nM) for 1, 4, 12, and 24 h, and surface PD‑L1 levels were quantified by flow cytometry using a non‑competing antibody. Surface PD‑L1 began to decline within 4 h of treatment, with maximal degradation (∼80% reduction) observed after 24 h (Figure 2c). A similar time course was observed in MDA‑MB‑231 cells (Figure S2D).

Confocal laser scanning microscopy further corroborated these findings. 4T1 cells treated with LA‑LYTAC^Ab^ (10 nM HS636^DBCO^ + 1000 nM LA^N3^) for 24 h were stained for PD‑L1 (red) and lysosomes (LysoTracker, green). Robust PD‑L1 internalization and extensive co‑localization with lysosomal compartments were observed in treated cells, whereas control cells exhibited predominantly membrane‑localized PD‑L1 signal (Figure 2d). Identical results were obtained in MDA‑MB‑231 cells (Figure S2A) and further confirmed in 4T1 cells (Figure S2B). To delineate the degradation pathway, we employed pharmacological inhibitors. MDA‑MB‑231 cells were treated with LA‑LYTAC^Ab^ (10 nM HS636^DBCO^ + 1000 nM LA^N3^) for 24 h in the presence of either MG132 (a proteasome inhibitor) or bafilomycin A1 (BAF, a lysosomal acidification inhibitor). Western blot analysis revealed that BAF completely blocked PD‑L1 degradation, whereas MG132 had no effect (Figure 2e), unequivocally establishing that LA‑LYTAC^Ab^ promotes PD‑L1 degradation through the lysosomal pathway, consistent with LAT1‑mediated endocytosis.

Finally, we validated the degradation potency using flow cytometry with a different anti‑PD‑L1 antibody (not HS636) to measure total cellular PD‑L1 levels after 24 h of treatment. 4T1 cells were treated with varying concentrations of LA^N3^ alone, HS636^DBCO^ alone, or the intact LA‑LYTAC^Ab^ complex. The results showed that LA‑LYTAC^Ab^ induced a concentration‑dependent reduction in total PD‑L1 levels, Neither the LAT1 ligand nor the antibody alone exhibited significant degradation activity (Figure 2f), further confirming that bivalent engagement of both PD‑L1 and LAT1 is required for efficient degradation. Collectively, these data demonstrate that LA‑LYTAC^Ab^ effectively mediates the degradation of PD‑L1 in TNBC cells via LAT1‑dependent endocytosis and lysosomal trafficking. The platform exhibits favorable potency and kinetics, with degradation proceeding through the lysosomal pathway. Given the critical role of PD‑L1 in tumor immune evasion and the limited internalization capacity of conventional PD‑L1 antibodies, this strategy offers a compelling approach to durably eliminate PD‑L1 from the tumor cell surface, with potential implications for cancer immunotherapy.

### 2.3 LA‐LYTAC^Cet^ Mediates Degradation of EGFR and Suppresses Downstream Signaling

To test the generality of our modular platform, we next targeted a mechanistically distinct oncogenic membrane protein: EGFR. Unlike PD‐L1, which primarily mediates immune checkpoint function, EGFR is a receptor tyrosine kinase that drives proliferation, survival, and metastasis through downstream MAPK and PI3K‐Akt pathways. We generated LA‐LYTAC^Cet^ by conjugating the LAT1 ligand LAN3 to the anti‐EGFR antibody cetuximab (Cet^DBCO^ + LA^N3^). HeLa cells, which express high levels of both LAT1 and EGFR, were used for this study [32, 33].

**FIGURE 3:**
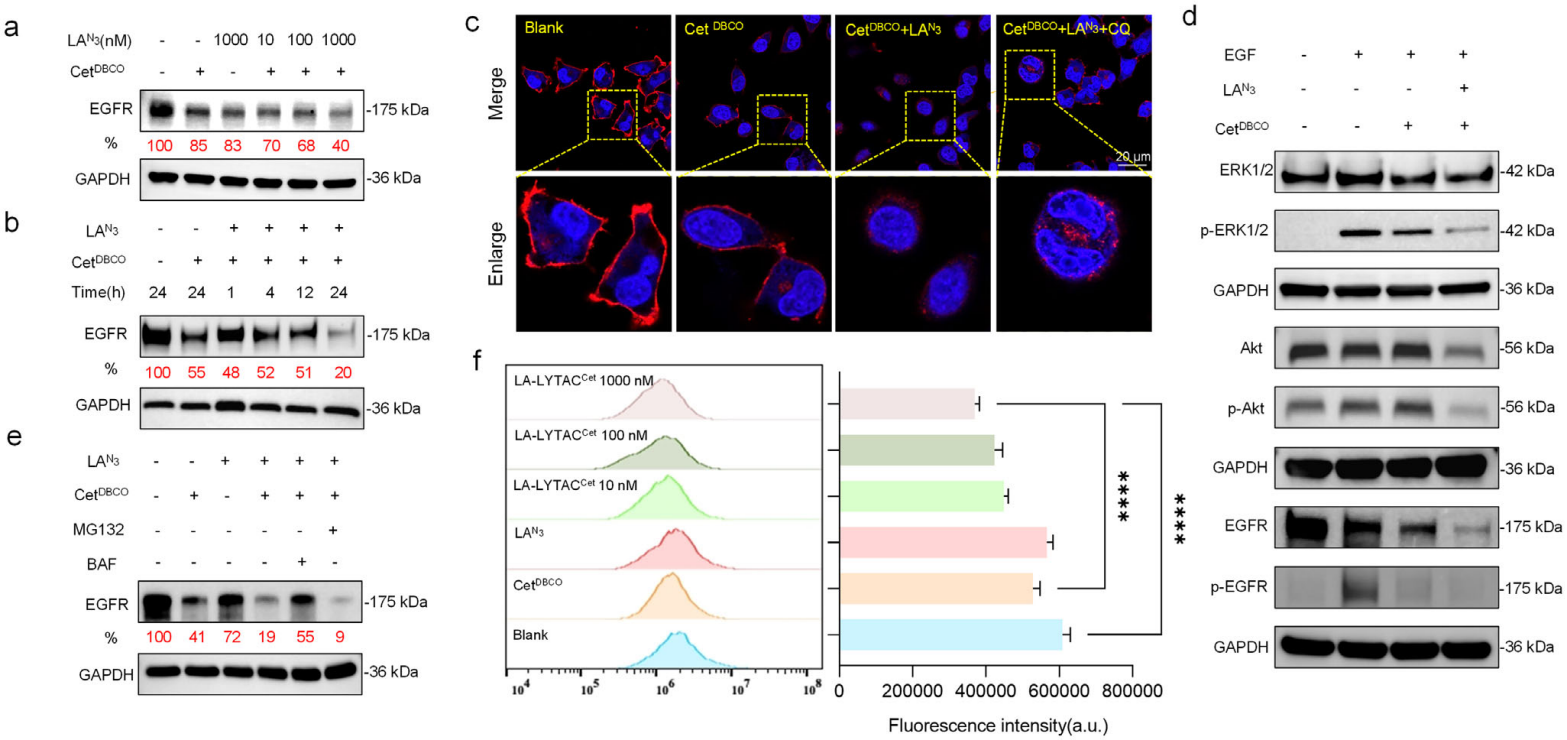
LA‐LYTAC^Ab^ promotes the degradation of EGFR in the HeLa cell line. (a) Western blot analysis of total EGFR levels in HeLa cells after treatment with 10 nM Cet^DBCO^ and 10 nM, 100 nM or 1000 nM LA^N3^ for 24 h. (b) Time course of EGFR degradation on the surface of HeLa cells after incubation with 10 nM Cet^DBCO^ and 1000 nM LA^N3^ for 1, 4, 12 and 24 h. (c) Confocal microscopy images of EGFR degradation in HeLa cells after treatment with 10 nM Cet^DBCO^ and 1000 nM LA^N3^ for 24 h. Scale bar: 20 µm. (d) Western blot detection of p‐ERK1/2, p‐Akt and p‐EGFR in HeLa cells after incubation with 10 nM Cet^DBCO^ and 1000 nM LA^N3^ for 24 h and stimulation with 100 ng/ml EGF for 20 min. (e) Western blot of EGFR degradation in HeLa cells after incubation with 10 nM Cet^DBCO^ and 1000 nM LA^N3^ for 24 h in the presence of 1 µM MG132 or 1 µM bafilomycin (BAF). (f) Flow cytometry analysis of EGFR degradation in HeLa cells after treatment with different concentrations of LA‐LYTAC^Cet^, LA^N3^ and Cet^DBCO^ (n = 3 per group).

Treatment of HeLa cells with a fixed concentration of Cet^DBCO^ (10 nM) and increasing LA^N3^ (10–1000 nM) for 24 h led to a dose‐dependent reduction in total EGFR protein, with near‐complete loss at 1000 nM LA^N3^ (Figure 3a). Neither component alone was effective, confirming the requirement for bivalent engagement, and equivalent results were obtained in 4T1 cells (Figure S4C). Surface EGFR levels, measured by flow cytometry with a non‐competing antibody, declined within 4 h of treatment with LA‐LYTAC^Cet^ (10 nM Cet^DBCO^ + 1000 nM LA^N3)^ and reached approximately 75% reduction by 24 h (Figure 3b); a similar time course was observed in 4T1 cells (Figure S4D). Confocal microscopy showed that LA‐LYTAC^Cet induced robust EGFR internalization, in contrast to the predominantly plasma membrane‐localized EGFR staining observed in control cells (Figure 3c; Figure S4A,B). The internalized EGFR signal strongly overlapped with LysoTracker‐positive acidic compartments. In addition, co‐staining with LAMP1, a lysosomal membrane marker, further confirmed lysosomal routing. Quantitative co‐localization analysis using Pearson's correlation coefficient showed significantly increased overlap between EGFR/LA‐LYTAC‐associated fluorescence and LAMP1‐positive compartments after LA‐LYTAC^Cet treatment, supporting lysosomal delivery of EGFR (Figure S5).

A key distinction of EGFR from PD‐L1 is its direct role in activating proliferative cascades. We therefore asked whether LA‐LYTAC^Cet^‐mediated degradation translates into pathway inhibition. HeLa cells treated with LA‐LYTAC^Cet^ for 24 h were stimulated with EGF (100 ng/mL, 20 min). Western blot analysis showed that LA‐LYTAC^Cet^ strongly suppressed basal and EGF‐induced phosphorylation of EGFR (p‐EGFR), ERK1/2 (p‐ERK1/2), and Akt (p‐Akt) (Figure 3d). Thus, degrading EGFR not only removes the receptor but also ablates its downstream signaling output [34]. Using pharmacological inhibitors, we found that bafilomycin A1 (BAF, a lysosomal acidification inhibitor) completely blocked EGFR degradation, whereas the proteasome inhibitor MG132 had no effect (Figure 3e), confirming that LA‐LYTAC^Cet^ operates through the lysosomal pathway. Flow cytometry quantification of surface EGFR after treatment with varying concentrations of LA‐LYTAC^Cet^, LA^N3^ alone, or Cet^DBCO^ alone demonstrated that only the intact chimera induced a concentration‐dependent reduction, with a DC50 in the low nanomolar range (Figure 3f); similar potency was observed in 4T1 cells (Figure S4E).

Collectively, these results establish that the LA‐LYTAC platform can be readily adapted to degrade a receptor tyrosine kinase (EGFR) with high potency, leading to functional suppression of downstream proliferative signaling. The consistent lysosomal mechanism across two distinct targets (PD‐L1 and EGFR) and two cell lines (4T1 and HeLa) further supports the modularity and broad applicability of the platform.

### 2.4 LA‐LYTAC^Ab^‐Mediated Degradation Requires LAT1

To confirm that the degradation activity of LA‐LYTAC chimeras depends on the LAT1 receptor, we knocked down *SLC7A5* (the gene encoding LAT1) using specific siRNA in three cell lines: MDA‐MB‐231 (human TNBC), HeLa (cervical cancer), and 4T1 (murine TNBC). Following knockdown, cells were treated with LA‐LYTAC^Ab^ (HS636^DBCO^ + LA^N3^) or LA‐LYTAC^Cet^ (Cet^DBCO^ + LA^N3^), and target protein levels were analyzed by Western blot. In MDA‐MB‐231 cells, PD‐L1 degradation induced by LA‐LYTAC^Ab^ was completely abrogated upon *SLC7A5* knockdown, whereas control siRNA had no effect (Figure S6A). Similarly, in HeLa cells, *SLC7A5* knockdown abolished EGFR degradation by LA‐LYTAC^Cet^ (Figure S6B). In 4T1 cells, PD‐L1 degradation was likewise dependent on *SLC7A5* expression (Figure S6C). Importantly, treatment with LAN3 alone or the antibody^DBCO^ conjugate alone did not affect target protein levels regardless of *SLC7A5* status, and *SLC7A5* knockdown itself did not alter basal PD‐L1 or EGFR expression. Furthermore, Western blot analysis confirmed that the expression level of LAT1 protein was efficiently reduced by siRNA, while treatment with LA‐LYTAC chimeras or their components did not affect LAT1 expression (Figure S6D). Collectively, these results demonstrate that LA‐LYTAC‐mediated degradation of both PD‐L1 and EGFR is strictly dependent on the presence of LAT1, confirming that the platform operates through the intended receptor‐mediated endocytosis pathway.

### 2.5 Small‐Molecule LA‐LYTACs Mediate Degradation of PD‐L1 and Integrins

**FIGURE 4:**
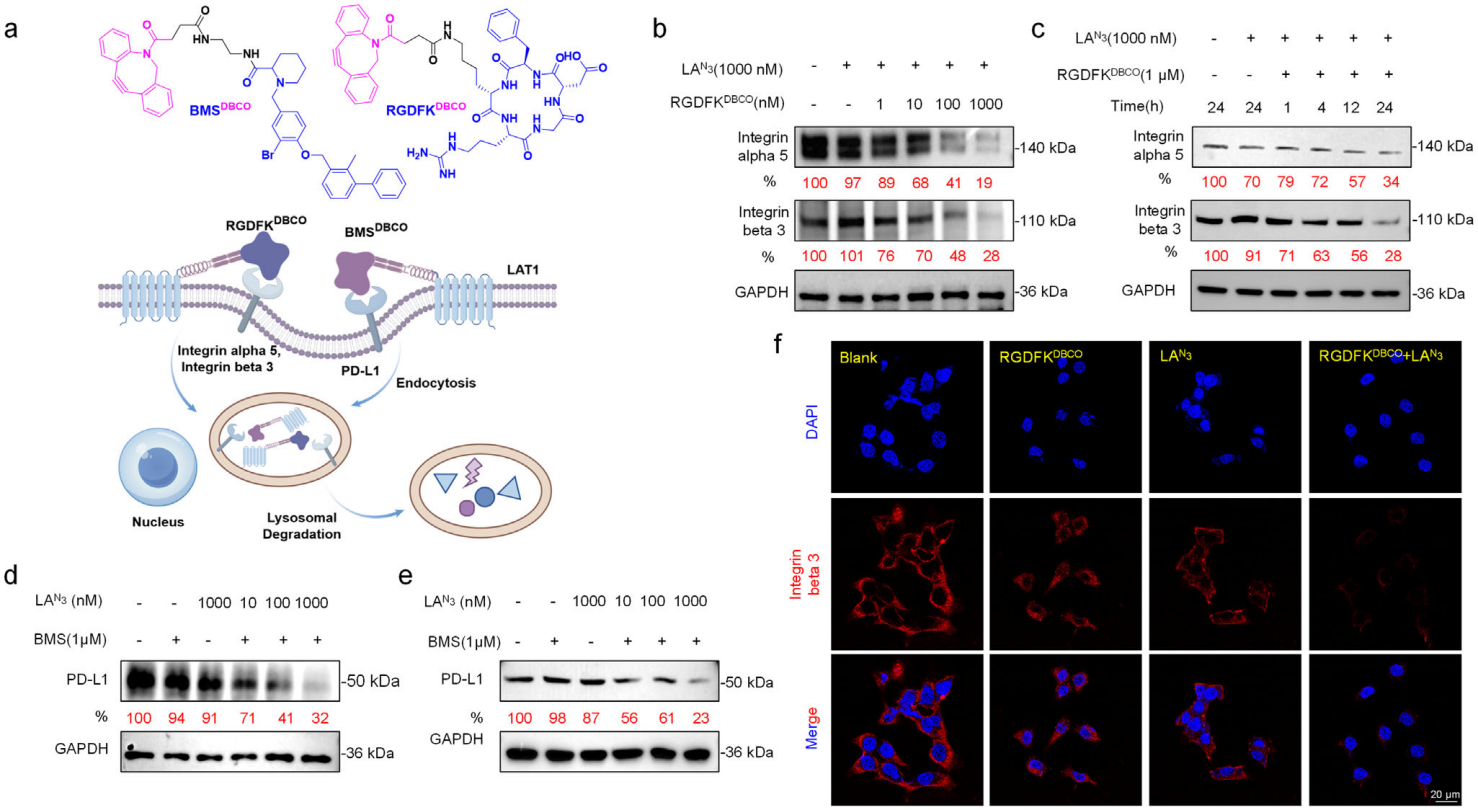
LA‐LYTAC promotes the degradation of PD‐L1 and integrins. (a) Schematic diagram of LA‐LYTAC‐mediated degradation of PD‐L1 and integrins. (b) Western blot analysis of changes in total integrin alpha 5 and integrin beta 3 levels in 4T1 cells after treatment with 1000 nM LAN3 and 10 nM, 100 nM or 1000 nM RGDFK^DBCO^ for 24 h. (c) Time course of degradation of surface integrins integrin alpha 5 and integrin beta 3 in 4T1 cells after incubation with 1000 nM RGDFK^DBCO^ and 1000 nM LA^N3^ for 1, 4, 12 and 24 h. (d) Western blot analysis of PD‐L1 levels in MDA‐MB‐231 cells after treatment with 1000 nM BMS^DBCO^ and 10 nM, 100 nM or 1000 nM LA^N3^ for 24 h. (e) Western blot analysis of PD‐L1 levels in 4T1 cells after treatment with 1000 nM BMS^DBCO^ and 10 nM, 100 nM or 1000 nM LA^N3^ for 24 h. (f) Confocal microscopy observation of integrin beta 3 degradation in 4T1 cells after treatment with 1000 nM RGDFK^DBCO^ and 1000 nM LA^N3^ for 24 h (*n* = 3 per group).

Having demonstrated that antibody‑based LA‑LYTAC effectively degrade PD‑L1 and EGFR, we next asked whether the platform could be extended to small‑molecule targeting moieties—a feature that would greatly simplify synthesis, reduce cost, and potentially improve tissue penetration. The modular design of LA‑LYTAC allows straightforward replacement of the target‑binding module, enabling rapid adaptation to diverse targets. To test this, we developed two distinct small‑molecule chimeras: one targeting integrin αvβ3 using the cyclic RGD peptide RGDFK (LA‑LYTAC^RGDFK^) [35], and another targeting PD‑L1 using the small‑molecule inhibitor BMS‑8 (LA‑LYTAC^BMS^) [36]. Both were conjugated to the same LAT1 ligand LA^N3^ via bioorthogonal chemistry (RGDFK^DBCO^ Figure S12 or BMS^DBCO^ Figure S13+ LA^N3^), as illustrated in Figure 4a.

We first evaluated integrin alpha 5 and integrin beta 3 in 4T1 murine TNBC cells. Treatment with a fixed concentration of LA^N3^ (1000 nm) and increasing RGDFK^DBCO^ (10–1000 nm) for 24 h led to a concentration‑dependent reduction in both integrin alpha 5 and integrin beta 3 protein levels, with maximal degradation at 1000 nM (Figure 4b). Neither component alone was effective, confirming the requirement for the intact chimera. Time‑course analysis of surface integrins by flow cytometry showed that integrin alpha 5 and integrin beta 3 levels began to decline within 4 h and reached a sustained reduction by 24 h (Figure 4c). Confocal microscopy further revealed that LA‑LYTAC^RGDFK^ induced robust internalization of integrin beta 3 and its co‑localization with LysoTracker‑positive lysosomes (Figure 4f); similar results were obtained for integrin alpha 5 (Figure S7). These data establish that a small‑peptide‑based LAT1 chimera efficiently delivers integrins to lysosomes.

To test a non‑peptidic small molecule, we conjugated BMS‑8 (a high‑affinity PD‑L1 inhibitor) to LA^N3^. Treatment of MDA‑MB‑231 human TNBC cells (Figure 4d) and 4T1 murine TNBC cells (Figure 4e) with a fixed concentration of BMS^DBCO^ (1000 nm) and increasing LA^N3^ (10–1000 nm) for 24 h resulted in a concentration‑dependent loss of PD‑L1 in both cell lines, with maximal degradation at 1000 nm LA^N3^. Neither BMS^DBCO^ nor LA^N3^ alone affected PD‑L1 levels. Notably, the efficacy was comparable between human and murine cells, demonstrating cross‑species functionality.

To further confirm that the observed biological activity was derived from the covalently linked LA‐LYTAC chimera, we performed additional purification and characterization using the BMS^DBCO^/LA^N3^ click reaction as a representative example. The crude click reaction product was purified by column chromatography and subsequently analyzed by mass spectrometry, which confirmed the expected molecular identity of the LA‐LYTAC^BMS^ conjugate. Analytical LC–MS comparison between the crude reaction mixture and the purified product showed that the desired LA‐LYTAC^BMS^ was already the predominant component in the crude mixture under our reaction conditions, with no substantial difference observed after purification (Figure S8). In addition, the crude and purified LA‐LYTAC^BMS^ samples exhibited comparable degradation activity in the cellular assay, indicating that the biological effect mainly resulted from the covalently formed LA‐LYTAC^BMS^ chimera rather than unreacted starting materials or other impurities (Figure S9). These results support the reliability of the click‐generated LA‐LYTAC products used in this study.

Collectively, these results demonstrate that the LA‑LYTAC platform readily accommodates small‑molecule targeting modules—including cyclic peptides and synthetic inhibitors—without loss of degradation activity. The ability to target two functionally distinct proteins (integrins and PD‑L1) using the same LAT1 ligand underscores the true modularity of this approach. Small‑molecule based degraders offer practical advantages over antibody‑based systems, including simplified synthesis, lower production costs, and potential for enhanced solid tumor penetration, thereby broadening the therapeutic scope of lysosome‑targeted degradation.

### 2.6 MD Simulations of LA‐LYTAC

**FIGURE 5:**
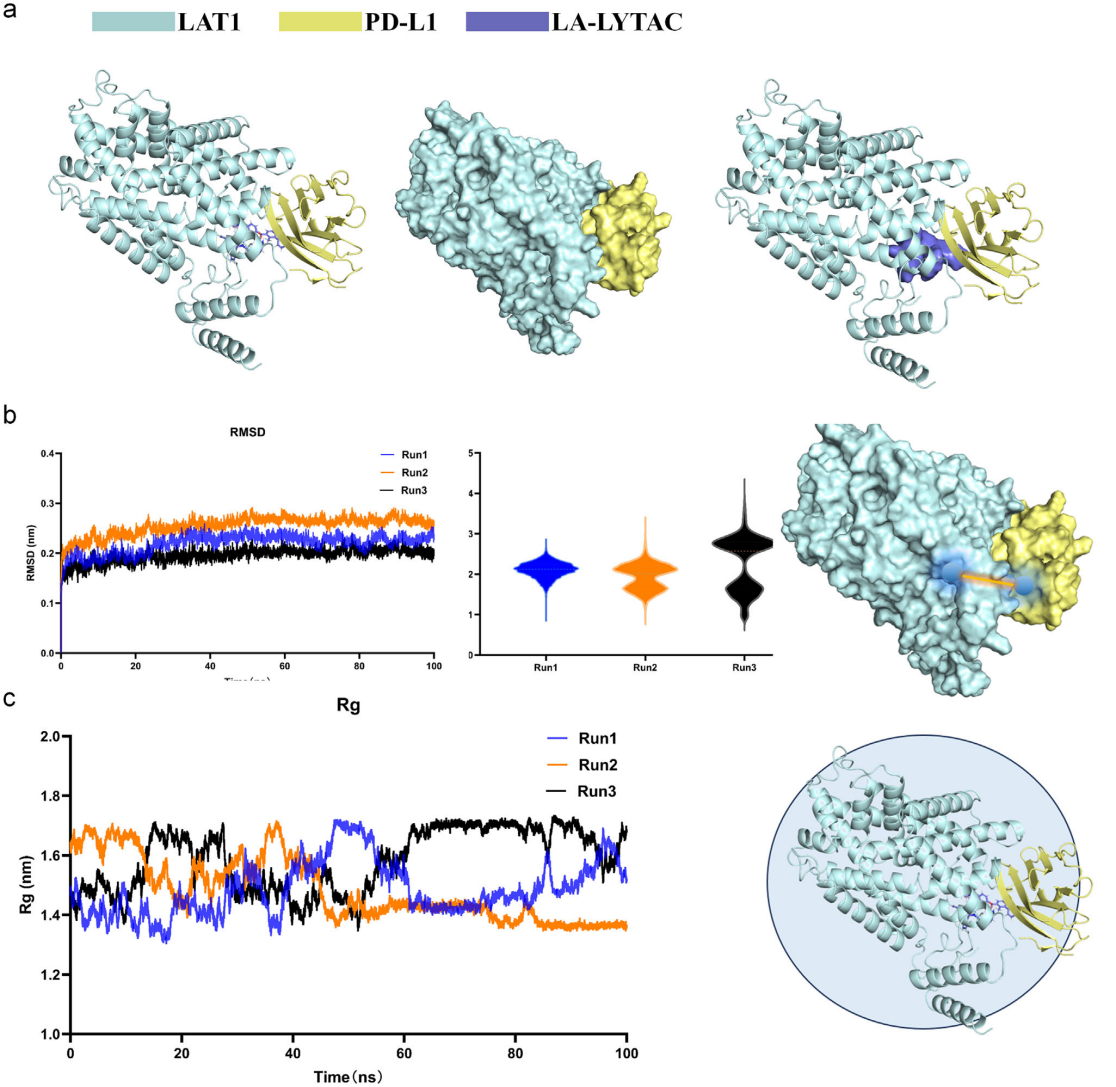
Comparative molecular dynamics analysis of the LA‐LYTAC complexes. Stable LAT1‐LA‐LYTAC‐PD‐L1 conformation (a) after MD simulations. RMSD profiles for LAT1‐LA‐LYTAC‐PD‐L1 across triplicate simulations. (b) Violin plots comparing centroid distances between LAT1 and PD‐L1 from triplicate MD simulations. (c) Comparative analysis of radius of gyration (Rg) distributions from triplicate MD simulations.

To further verify the ability of the bifunctional molecule to induce the formation of a ternary complex between LAT1 and PD‐L1, molecular dynamics simulations were performed in this study. The results showed that the LAT1‑Compound‑PD‐L1 ternary complex maintained good structural integrity throughout the MD simulation (Figure 5a). Root Mean Square Deviation (RMSD) is a key indicator for evaluating structural fluctuation and stability of the system. Analysis revealed that the average RMSD of the whole complex was 0.2230 ± 0.0172 nm (Figure 5b). The low RMSD value and small standard error indicated that the system rapidly reached equilibrium within 100 ns of simulation and retained high conformational stability. The RMSD of the complex quickly stabilized at approximately 0.22 nm at the early stage of simulation without obvious large fluctuations, suggesting a high‑affinity binding interface that effectively prevented backbone distortion or ligand dissociation. Such a low RMSD feature directly demonstrated the high stability of the system, indicating that the bifunctional molecule can efficiently stabilize the binding between LAT1 and PD‐L1. Furthermore, the interprotein centroid distance was calculated. The results indicated tight molecular interactions within the complex, with an average centroid distance of 2.1033 ± 0.0917 nm (Figure 5b). Radius of Gyration (Rg) is commonly used to evaluate the compactness of protein structures. The simulation results showed that the average Rg of the complex was 1.5178 ± 0.0334 (Figure 5c). The low standard error suggested that the protein remained highly compact and structurally uniform during the entire simulation, without obvious unfolding or loosening. The stable Rg further confirmed that the binding of the bifunctional molecule to target proteins helps maintain the native folded conformation, reduce conformational drift caused by external disturbances, and thus enhance the overall structural stability of the ternary complex.

### 2.7 LA‐LYTAC Enhances Antitumor Immunity Through PD‐L1 Degradation

**FIGURE 6:**
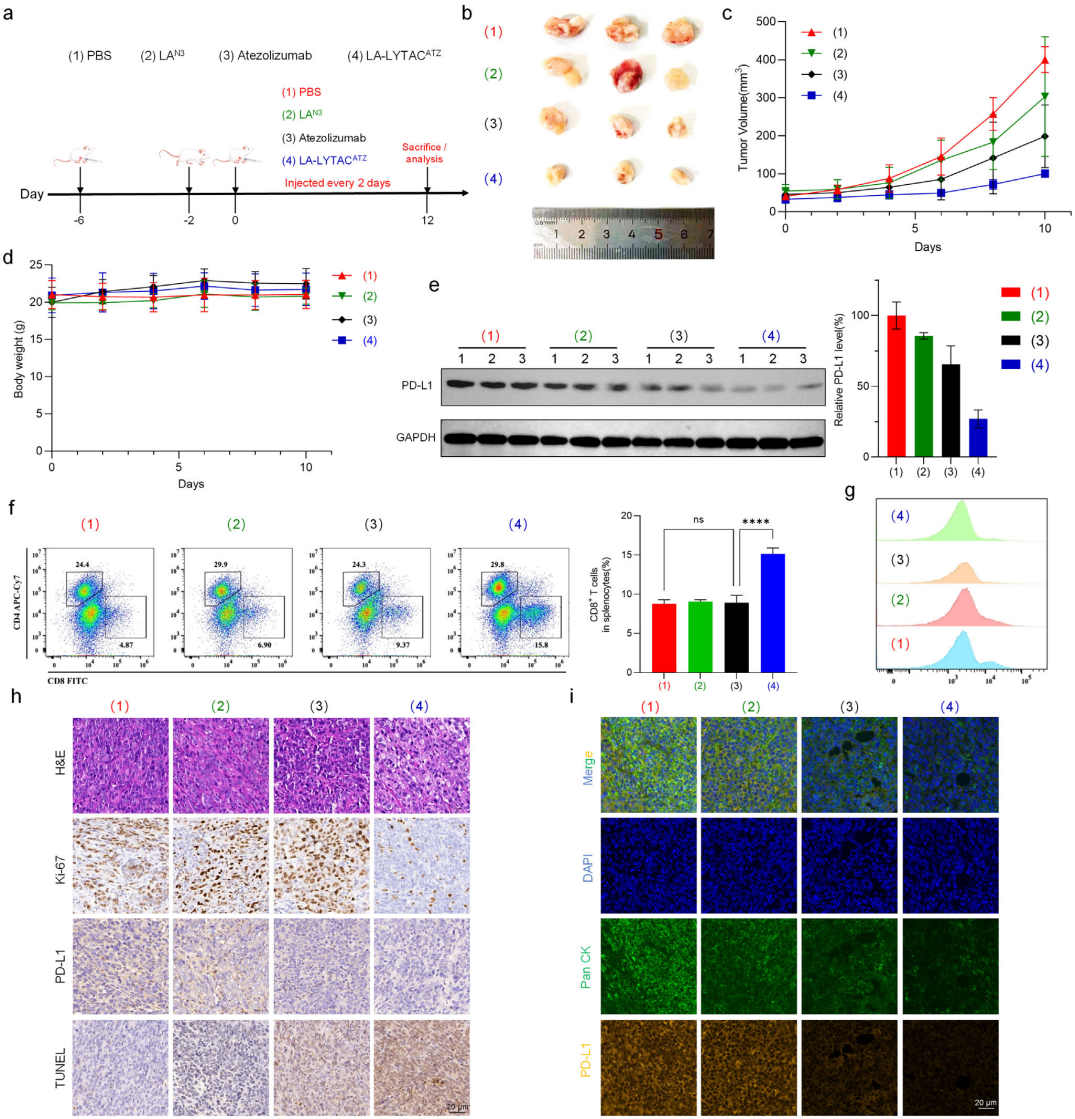
The inhibitory effect of LA‐LYTAC on mouse tumors. (a) Schematic diagram of LA‐LYTAC treatment in Balb/c tumor‐bearing mice model. 4T1 cells were subcutaneously injected into mice. Drug administration (0.5 mg/kg LA‐LYTAC^ATZ^) was performed when the tumor size reached approximately 100 mm^3^. (b) Photographs of dissected tumor tissues and comparison of tumor volumes on day 12 (*n* = 3 per group). (c) Quantification of tumor weight from each group (*n* = 3 per group). (d) Body weight of mice in each group was measured every two days (*n* = 3 per group). (e) Western blot analysis of PD‐L1 expression in tumor tissues (*n* = 3 per group). (f) Flow cytometry analysis of CD8^+^ T cells in the spleen of mice on day 12 after different treatments (*n* = 3 per group). (g) Flow cytometry analysis of PD‐L1 levels in tumor tissues of different treatment groups (*n* = 3 per group). (h) Representative immunohistochemical staining results of H&E, Ki67, PD‐L1, and TUNEL in subcutaneous tumor tissues of mice in different treatment groups. Scale bar, 20 µm. (i) Representative immunohistochemical staining results of Pan CK and PD‐L1 in subcutaneous tumor tissues of mice in different treatment groups. Scale bar, 20 µm.

To evaluate the therapeutic potential of LA‐LYTAC, we employed a syngeneic mouse model using 4T1 murine triple‐negative breast cancer (TNBC) cells, which express high levels of LAT1 and PD‐L1. BALB/c mice were subcutaneously inoculated with 4T1 cells, and when tumors reached approximately 100 mm^3^, mice were treated with LA‐LYTAC^ATZ^ (0.5 mg ·kg^−^^1^, intraperitoneal injection every two days for three doses), which consists of the anti‐PD‐L1 antibody atezolizumab conjugated to the LAT1 ligand LA^N3^. Control groups received vehicle, atezolizumab alone, or LA^N3^ alone (Figure 6a).

LA‐LYTAC^ATZ^ treatment significantly suppressed tumor growth compared to all control groups. Tumor volumes were monitored every two days; by day 12, vehicle‐treated tumors reached a mean volume of approximately 1200 mm^3^, whereas LA‐LYTAC^ATZ^ treated tumors were only about 400 mm^3^, representing a greater than 60% reduction (Figure 6c). Representative tumor photographs and excised tumor weights at day 12 further confirmed the robust antitumor efficacy (Figure 6b). Importantly, no significant body weight loss was observed in any treatment group, indicating that LA‐LYTAC^ATZ^ was well tolerated at the administered dose (Figure 6d).

To confirm target engagement and degradation in vivo, we analyzed PD‐L1 expression in excised tumor tissues by Western blot. As shown in Figure 6e, PD‐L1 protein levels were markedly reduced in tumors from LA‐LYTAC^ATZ^‐treated mice compared to vehicle, atezolizumab, or LA^N3^ controls. Quantitative analysis revealed a greater than 80% reduction in PD‐L1 expression (*p* < 0.001), demonstrating that LA‐LYTAC^ATZ^ effectively degrades PD‐L1 in the tumor microenvironment.

We further validated PD‐L1 reduction by flow cytometry analysis of tumor tissues. Consistent with the Western blot data, tumors from LA‐LYTAC^ATZ^‐treated mice exhibited significantly reduced PD‐L1 levels on the surface of tumor cells compared to control groups (Figure 6g). This reduction was specific to tumor cells, as PD‐L1 expression on immune cell subsets was minimally affected. Immunohistochemical (IHC) analysis of PD‐L1 in tumor tissues confirmed the reduction at the protein level, with LA‐LYTAC^ATZ^‐treated tumors showing minimal PD‐L1 staining compared to control groups (Figure 6h, Figure S10). Double staining for pan‐cytokeratin (Pan CK, a tumor cell marker) and PD‐L1 further confirmed that PD‐L1 expression was specifically reduced on tumor cells following LA‐LYTAC^ATZ^ treatment (Figure 6i, Figure S11).

Given the central role of PD‐L1 in immune suppression, we next investigated whether PD‐L1 degradation translated to enhanced antitumor immunity. Spleens and tumors were harvested at day 12, and CD8^+^ T cell populations were analyzed by flow cytometry. As shown in Figure 6f, LA‐LYTAC^ATZ^ treatment resulted in a significant increase in the frequency and absolute number of CD8^+^ T cells within the spleen, indicating systemic immune activation. More importantly, analysis of tumor‐infiltrating lymphocytes revealed a marked increase in CD8^+^ T cell infiltration in LA‐LYTAC^ATZ^‐treated tumors compared to control groups (Figure 6i). These findings suggest that LA‐LYTAC^ATZ^‐mediated PD‐L1 degradation relieves immune suppression, enabling enhanced T cell infiltration and activation.

Collectively, these in vivo data demonstrate that LA‐LYTAC^ATZ^ effectively degrades PD‐L1 in the tumor microenvironment, leading to enhanced CD8^+^ T cell infiltration, reduced tumor proliferation, increased apoptosis, and significant suppression of tumor growth in a syngeneic TNBC model. The favorable safety profile, combined with the robust antitumor activity, positions LA‐LYTAC as a promising therapeutic strategy for PD‐L1‐driven malignancies.

## 3 Discussion

Targeted protein degradation has expanded beyond the ubiquitin–proteasome system to the endo‐lysosomal pathway, providing a promising strategy for eliminating extracellular and transmembrane proteins. Most existing lysosome‐targeting approaches, including classical LYTACs, rely on a limited number of endogenous lysosome‐targeting receptors, such as CI‐M6PR and ASGPR. However, the variable expression patterns of these receptors across different cell types and tissues may restrict the broader applicability of these strategies. Recent GLUT1‐mediated LYTAC [37] studies have demonstrated that nutrient transporters can also be exploited to promote lysosomal delivery and degradation of extracellular or membrane‐associated proteins, highlighting transporter‐mediated trafficking as an alternative route beyond conventional lysosome‐targeting receptors.

In this context, our LAT1‐directed LA‐LYTAC platform provides a distinct and complementary nutrient transporter‐mediated degradation strategy. LAT1 is frequently upregulated in diverse cancers and plays a critical role in transporting large neutral amino acids to support tumor growth, making it an attractive tumor‐associated receptor for lysosomal trafficking. Unlike conventional LYTAC systems that often require bulky glycopolymers, multivalent glycans, or antibody‐based lysosomal shuttles, our approach repurposes a minimal amino‐acid‐derived ligand as the LAT1‐recognition handle. Specifically, the use of a single phenylalanine‐derived ligand enables a compact molecular design with high synthetic modularity, facile derivatization, and broad compatibility with different conjugation formats, including antibody‐based and small‐molecule‐based degraders. This minimalist design may also offer potential advantages in terms of synthetic simplicity, reduced structural complexity, and improved developability. Thus, while GLUT1‐mediated LYTACs have established an important precedent for transporter‐mediated lysosomal degradation, the LAT1‐based LA‐LYTAC system described here expands the repertoire of nutrient transporter‐directed degradation platforms and offers an alternative modular strategy for targeting extracellular and membrane proteins, particularly in LAT1‐overexpressing tumor contexts.

Using this single‑amino‑acid handle, we developed a modular platform (LA‑LYTAC) that conjugates the LAT1 ligand to various warheads (antibodies or small molecules) against PD‑L1, EGFR, and integrins. The resulting chimeras achieved potent, concentration‑dependent degradation in multiple cancer cell lines via a lysosomal mechanism strictly dependent on LAT1. Notably, EGFR degradation suppressed downstream MAPK and PI3K‑Akt signaling [34], and PD‑L1 degradation in a syngeneic TNBC mouse model reduced tumor growth by >60%, enhanced CD8^+^ T cell infiltration, and showed no overt toxicity.

The LA‑LYTAC platform offers three key advantages over existing strategies. First, LAT1 is broadly overexpressed across cancers, enabling consistent target degradation in multiple tumor types. Second, the high‑capacity recycling of LAT1 reduces susceptibility to the “hook effect” over a wide concentration range. Third, the modular “one‑to‑many” design allows rapid conjugation to various warheads via a simple “mix‑and‑click” workflow, eliminating complex protein engineering or glycan synthesis. To our knowledge, this is the first lysosome‑targeting degrader that employs a single, natural‑derived amino acid as the receptor‑engaging element.

Mechanistic studies confirmed that LA‑LYTAC‑mediated degradation occurs via the endo‑lysosomal pathway and depends on LAT1 function. Future designs could incorporate pH‑sensitive or enzymatically cleavable linkers to enhance degradation potency and LAT1 recycling. Limitations include basal LAT1 expression in some normal tissues (e.g., blood‑brain barrier), necessitating careful on‑target, off‑tumor toxicity assessment. Additionally, linker chemistry and in vivo pharmacokinetics require optimization for each conjugate. Nevertheless, the extreme simplicity of the amino‑acid handle facilitates rapid iterative optimization.

It should be noted that the in vivo safety evaluation in the present study was preliminary. Although no obvious body weight loss was observed under the tested treatment conditions, body weight monitoring alone is insufficient to establish a comprehensive safety profile. In this proof‐of‐concept study, we primarily focused on evaluating the antitumor efficacy of LA‐LYTACs, and systematic toxicological assessments, including histopathological examination of major organs, serum biochemical analysis, and evaluation of potential immune‐related adverse effects, were not performed. Therefore, the current data should be interpreted as an initial indication of tolerability rather than definitive evidence of safety. Future studies will be required to comprehensively characterize the safety profile of LA‐LYTACs, including organ toxicity, hepatic and renal function, hematological parameters, inflammatory cytokine responses, and potential immunogenicity after repeated administration.

An important consideration for the modular application of LA‐LYTACs is whether conjugation of a bulky cargo, particularly an antibody, affects the ability of the amino‐acid‐derived handle to engage LAT1. Although the LAT1 ligand used in this study is structurally small, attachment of a large biomacromolecule could in principle alter local presentation, reduce apparent binding affinity, or affect transporter‐mediated internalization because of steric hindrance or changes in conjugation geometry. In the present design, however, antibody‐based LA‐LYTACs retained efficient cellular uptake, lysosomal trafficking, and target degradation activity, suggesting that LAT1 engagement is tolerated despite the presence of a bulky antibody payload. Nevertheless, we acknowledge that the current study primarily evaluates functional degradation outcomes rather than directly quantifying LAT1‐binding affinity after conjugation to different cargoes. Future studies involving systematic affinity measurements, transporter competition assays, and comparison of different linker lengths, conjugation sites, and cargo sizes will be important to further optimize ligand presentation and define the structural parameters required for efficient LAT1‐mediated lysosomal delivery.

In summary, we have established LAT1 as a powerful lysosome‑targeting receptor and demonstrated a modular, synthetically accessible degradation platform driven by a single‑amino‑acid handle. This minimalist design provides a versatile new tool for chemical biology and opens the door to a new generation of lysosome‑targeting degraders with improved drug‑like properties.

## 4 Conclusions

In summary, we have repurposed LAT1 as a tumor‑selective lysosomal targeting receptor using a single phenylalanine derivative as the minimal chemical handle. This minimalist design enables a modular, plug‑and‑play platform (LA‑LYTAC) that can be rapidly conjugated to various warheads for targeted degradation of oncogenic membrane proteins. The approach is synthetically accessible, modular, and broadly applicable, offering a streamlined alternative to existing lysosome‑targeting technologies.

## Author Contributions

**Liquan Zhu**: conceptualization, methodology, writing – original draft, data curation, formal analysis. **Ke Liu**: conceptualization, methodology, data curation, formal analysis, writing – original draft. **Haotian Liu**: conceptualization, methodology, data curation, formal analysis, writing – original draft. **Chaoqi He**: methodology, formal analysis, data curation. **Xiaozhen Liu**: methodology, formal analysis, data curation. **Xin Zeng**: methodology, formal analysis. **Misha Mao**: validation, software, data curation. **Yuxiao Mu**: data curation, formal analysis. **Ying Li**: conceptualization, investigation, formal analysis. **Qinghui Zheng**: data curation, formal analysis. **Hongchao Tang**: data curation, formal analysis. **Da Qian**: conceptualization, writing – review and editing, funding acquisition, visualization. **Xuli Meng**: conceptualization, funding acquisition, supervision, resources, project administration, writing – review and editing.

## Conflicts of Interest

The authors declare no conflicts of interest.

## Supporting information




**Supporting File 1**: advs76862‐sup‐0001‐SuppMat.docx

## Data Availability

The data that support the findings of this study are available from the corresponding author upon reasonable request.
