# Supplementary material for: A Single‐Amino‐Acid Ligand for LAT1: A Minimalist and Modular Platform for Lysosome‐Targeted Degradation of Membrane Proteins
Source: Adv Sci (Weinh). 2026 Aug 3:e76862. Online ahead of print. doi: 10.1002/advs.76862 (PMC13430922; doi:10.1002/advs.76862)
Supplement: Supplementary file 1 — Supporting File 1: advs76862‐sup‐0001‐SuppMat.docx [file ADVS-9999-e76862-s001.docx]

**A Single-Amino-Acid Ligand for LAT1: A Minimalist and Modular Platform for Lysosome-Targeted Degradation of Membrane Proteins**

Liquan Zhu^1, 2†^, Ke Liu^1, 2†^ Haotian Liu^1, 2^,Chaoqi He^1, 2^, Xiaozhen Liu^1, 2^, Xin Zeng^1, 2^, Misha Mao^1, 2^, Yuxiao Mu^1, 2^, Ying Li^1, 2^, Qinghui Zheng^1, 2^, Hongchao Tang^1, 2^ , Da Qian^3*^ , Xuli Meng^1, 2*^

^1^ Department of Breast Surgery, General Surgery, Cancer Center, Zhejiang Provincial People’s Hospital, Affiliated People’s Hospital, Hangzhou Medical College, Hangzhou, Zhejiang, 310014, China

^2^ Key Laboratory for Diagnosis and Treatment of Upper Limb Edema and Stasis of Breast Cancer, Hangzhou, 310014, Zhejiang, China

^3^ Central Laboratory, Changshu Hospital Affiliated to Soochow University, Changshu No.1 People's Hospital, Changshu 215500, Jiangsu, China

Liquan Zhu †, Ke Liu †, contributed equally to this work

Correspondence: Da Qian [(qianda0215@suda.edu.cn),](mailto:(qianda0215@suda.edu.cn),) Xuli Meng [(mxlmial@126.com)](mailto:(mxlmial@126.com))

**content**

[S1 Materials & Methods 3](#_Toc232947820)

[S1.1 General information 3](#_Toc232947821)

[S1.2 Cell culture 3](#_Toc232947822)

[S1.3 Preparation and purification of LA-LYTAC 3](#_Toc232947823)

[S1.3.1 DBCO modification of antibodies (e.g., Cet, HS636, SLG) 3](#_Toc232947824)

[S1.3.2 Conjugation via click chemistry to form LA-LYTAC 3](#_Toc232947825)

[S1.4 Neutravidin uptake experiment with LA^Biotin^ 3](#_Toc232947826)

[S1.5 Neutravidin degradation experiment 4](#_Toc232947827)

[S1.6 Protein degradation analysis by western blotting (WB) 4](#_Toc232947828)

[S1.7 Immunofluorescent analysis 4](#_Toc232947829)

[S1.8 Construction and transfection of small interfering RNAs (siRNAs) 4](#_Toc232947830)

[S1.9 In vivo animal studies 5](#_Toc232947831)

[S1.10 Details or analysis of molecular dynamics simulations 5](#_Toc232947832)

[S2 Supplementary Text 6](#_Toc232947833)

[S2.1 LA-LYTAC^Biotin^ mediates the internalization and degradation of NA650 6](#_Toc232947834)

[S2.2 LA-LYTAC^Ab^ promotes PD-L1 degradation in MDA-MB-231 and 4T1 cell lines 7](#_Toc232947835)

[S2.3 LA-LYTAC^Ab^ promotes the degradation of EGFR in HeLa and 4T1 cell lines 8](#_Toc232947836)

[S2.4 LA-LYTAC^Ab^ promotes protein degradation in a LAT1 (SLC7A5)-dependent manner 10](#_Toc232947837)

[S2.5 Confocal microscopy observation of integrin alpha 5 degradation in 4T1 cells 11](#_Toc232947838)

[S2.6 Representative whole and magnified views of TUNEL-stained tumor 13](#_Toc232947839)

[S2.7 Immunofluorescence analysis of cell surface PD-L1 protein 14](#_Toc232947840)

[S2.8 Mass spectrum of RGDFK^DBCO^ 15](#_Toc232947841)

[S2.9 Mass spectrum of BMS^DBCO^ 15](#_Toc232947842)

[S2.10 Mass spectrum of LA^Biotin^ 16](#_Toc232947843)

[S2.11 NMR and Mass Spectrum of LA^N3^ 17](#_Toc232947844)

[S3 Tables 19](#_Toc232947845)

# S1 Materials & Methods

## S1.1 General information

RPMI 1640 (Cellmax), DMEM (Hyclone) and Fetal Bovine Serum (FBS) were purchased from qualified vendors. NeutrAvidin Protein, DyLightTM 650 (NA650) was purchased from Invitrogen (Catalog No:84607, 22832). Human IgG was purchased from Sigma-Aldrich (Catalog No: I8640). DBCO-PEG_4_-NHS (Catalog No: BDC-7) and Cyanine 5-DBCO (Catalog No: BDC-11) was purchased from Confluore (China). Lyso­Tracker™Green DND-26 and Hoechst 33342 were purchased fromInvitrogen. chloroquine (Sigma-Aldrich), Bafilomycin A1 (Solarbio) and MG-132 (Sigma-Aldrich) were purchased from the indicated suppliers. All other materials and reagents were purchased from commercial sources and used as received, unless stated otherwise.

## S1.2 Cell culture

All cell lines were donated by Zhejiang Provincial People’s Hospital. Cells were cultured in T25 flasks under 5 % CO2 at 37 ◦C. MDA-MB-231 cells was cultured in complete growth medium (RPMI 1640 supplemented with 10 % FBS and 1 % penicillin/streptomycin (Sal­orbio)). HeLa, 4T1 cells were cultured in complete growth medium (DMEM supplemented with 10 % FBS and 1 % penicillin/streptomycin).

## S1.3 Preparation and purification of LA-LYTAC

### S1.3.1 DBCO modification of antibodies (e.g., Cet, HS636, SLG)

Antibodies were first buffer-exchanged into PBS (pH 7.4) using 30 kDa molecular weight cut-off (MWCO) filters to remove interfering agents.The antibody solution (1 mg/mL in PBS) was mixed with an equal volume of 100 mM NaHCO3 buffer (pH 8.5) to adjust the reaction pH. DBCO-PEG_4_-NHS ester (dissolved in DMSO) was added to the antibody solution at a molar ratio of 10:1 (DBCO:Antibody). The reaction mixture was gently stirred at 4 ◦C for 12 h. The reaction was quenched by adding Tris-HCl buffer (pH 8.0) to a final concentration of 20 mM. The DBCO-modified antibody (Ab^DBCO^) was purified from unreacted reagents using Zeba™ Spin Desalting Columns (7K MWCO, Thermo Scientific) pre-equilibrated with PBS. The final concentration was determined by Nanodrop, and the product was stored at 4 ◦C for immediate use or at −20 ◦C for long-term storage.

### S1.3.2 Conjugation via click chemistry to form LA-LYTAC

The purified DBCO-modified antibody (Ab^DBCO^) was reacted with a 10-fold molar excess of the azide-functionalized LAT1 analog (LA^N3^) in PBS (pH 7.4). The conjugation reaction was carried out at room temperature for 4 h with gentle agitation.The resulting LA-LYTAC was typically used directly for cellular and in vivo experiments without further purification, as the click reaction proceeds with high efficiency. For analytical validation, the crude LA-LYTAC mixture was characterized by SDS-PAGE to confirm conjugate formation and assess purity.

## S1.4 Neutravidin uptake experiment with LA^Biotin^

Flow cytometry analysis: HeLa and MDA-MB-231 cells were plated in a 6-well plate before the experiment with~80 % confluency. After 1 day, 50 nM NA650 and 1000 nM LA^Biotin^ were added sequentially. Cells were further incubated at 37 ◦C for different time periods. Then cells were washed with PBS three times to remove extracellular NA650, detached and transferred to a Falcon tube before flow cytometry analysis (Agilent NovoCyte Quanteon). Median fluorescence intensity (MFI) of each cell population was calculated containing at least 10,000 live cells.

## S1.5 Neutravidin degradation experiment

HeLa and MDA-MB-231 cells were plated in a 6-well plate before the experiment with~80 % confluency. After 1 d, 50 nM NA650 and 1000 nM LA^Biotin^ were added sequentially. Cells were further incubated at 37 ◦C for different time periods. Then cells were washed with PBS three times to remove extracellular NA650, and cultured to fresh medium for another 24 h. Then cells were washed with PBS three times to remove extracellular NA650, detached and transferred to a Falcon tube before flow cytometry analysis. Median fluorescence intensity (MFI) of each cell population was calculated containing at least 10,000 live cells.

## S1.6 Protein degradation analysis by western blotting (WB)

Cells were seeded in 12-well plates to 70–80 % confluency and treated with different concentrations of LA-LYTAC in complete growth medium with indicated amount and time. For inhibitor-based assay, cells were pretreated with lysosomal inhibitors, bafilomycin A1 and chloroquine, respectively. For EGF stimulation, cells were washed and further treated with EGF (100 ng/mL) for 20 min at 37 ◦C. After incubation, cells were lysed in the presence of 1 mM protease inhibitor (Beyotime) and collected. The concentration of the protein lysate was determined by BCA assay. Next, equal amounts of different cell lysates were resolved onto a 10 % SDS-PAGE gel, followed by transfer to a PVDF membrane. The resulting membrane was then blocked with 5 % fat-free milk in TBST 5 % for 1.5 h at room temperature with shaking. After washing with TBST, the membrane was incubated overnight with the corresponding primary antibody at 4 ◦C and washed three times with TBST. Then, the membrane was incubated with the respective secondary antibody for 1 h at room temperature and washed three times with TBST, and finally imaged by using e-BLOT imaging system. The band intensities of WB were quantified by software Image J.

## S1.7 Immunofluorescent analysis

Cells were seeded in glass bottom-confocal dish with 70 % confluency. The next day, cells were incubated with 10 nM Cet^DBCO^, HS636^DBCO^ and LA^N3^ or controls for 24 h. Cells were washed, fixed with 5 % formaldehyde in PBS for 15 min at 37 ◦C and permeabilized with 0.1 % Triton X-100 in PBS for 15 min at 37 ◦C. After washing with PBS three times, cells were blocked in 3 % BSA in PBS for 1 h at room temperature and washed three times with PBS. Cells were incubated with the related primary antibody overnight at 4 ◦C and washed three times with PBS. Finally, cells were incubated with the secondary antibody (Alexa Fluor 647 anti-Rabbit) for 1.5h at room temperature (avoid light), washed three times with PBS and stained with Hoechst 33,342 for 10 min. After washing with PBS, cells were then imaged using CLSM (Leica, DMi8).

## S1.8 Construction and transfection of small interfering RNAs (siRNAs)

The sense and antisense human LAT1R siRNAs (siLAT1R) (5′- GAGGAUGGAAUUACUUGAATT-3′ and 5′ - UUCAAGUAAUUCCAUCCUCTT-3′, respectively) and sense and antisense mouse LAT1R siRNAs (siLAT1R) (5′- GGAUCGAGCUGCUCAUCAUTT-3′ and 5′ - AUGAUGAGCAGCUCGAUCCTT-3′, respectively) used in this experiment were synthesized by Shanghai Jima Pharmaceutical Technology Co., Ltd.(Shanghai, China). Control non-silencing siRNAs were also obtained from Shanghai Jima Pharmaceutical Technology Co., Ltd. (Silencer Negative Control siRNA, siControl). Then the siRNA duplexes were transfected to MDA-MB-231 、 HeLa and 4T1 using Lipofectamine™ 2000 (Thermo Fisher Scientific) according to the manufacturer’s protocol. After 1 d of transfection, cells were further used to analyze the PD-L1 and EGFR degradation efficiency.

## S1.9 In vivo animal studies

Female BALB/c mice (3–4 weeks old) were purchased from Gem-Pharmatech Co., Ltd. All mice were housed under specific pathogen-free conditions, with constant temperature at 24 ◦C, 55–65 % relative humidity and 12–12 h light–dark cycle. All the experimental procedures were conducted in accordance with guidelines approved by the animal ethics committee of the Zhejiang Provincial People’s Hospital and the project licenses are 20251119857004. For tumor inhibition studies, mouse 4T1 cells (1 × 10^6^ cells in 50 μl PBS) were inoculated subcutaneously into the right flank of BALB/c mice. After 5–7 days, mice were treated s.c. with PBS and 0.5 mg kg^−1^ LA-LYTAC at the indicated time points. Tumor growth and body weight were monitored every 2 days. Tumor volume was calculated using the formula: π/6 × tumor length × (tumor width)^2^. At the study endpoint, mice were euthanized, and tumor tissues were collected for flow cytometry analysis of tumor-infiltrating immune cells and for Western blot analysis of PD-L1 expression. Harvested tumors were fixed in 4 % paraformaldehyde (Servicebio, G1101-500 ml) for 24–48 h at room temperature, processed through graded ethanol/xylene, and embedded in paraffin. Sections (5 μm) were cut using a rotary microtome (Leica RM2235), mounted on microscope slides (CITOTEST,80313-7161-16) stained with hematoxylin and eosin and imaged using PANNORAMIC SCAN II (3DHISTECH). All procedures were approved by the Committee on the Ethics of Animal Experiments of Zhejiang Provincial People’s Hospital.

## S1.10 Details or analysis of molecular dynamics simulations

The protein structure was retrievedfrom the UniProt database and predicted using AlphaFold 3. The protein ID of PD-L1 is Q9NZQ7, and the protein ID of LAT1 is Q01650. Gromacs2022.3 software was used for moleculardynamics simulation. For small molecule preprocessing, AmberTools22 is used toadd GAFF force field to small molecules, while Gaussian 16W is used tohydrogenate small molecules and calculate RESP potential. Potential data willbe added to the topology file of molecular dynamics system. The simulationconditions were carried out at static temperature of 300K and atmosphericpressure (1 Bar). Amber99sb-ildn was used as force field, water molecules wereused as solvent (Tip3p water model), and the total charge of the simulationsystem was neutralized by adding an 3 Na+ ions. The simulation system adoptsthe steepest descent method to minimize the energy, and then carries out theisothermal isovolumic ensemble (NVT) equilibrium and isothermal isobaricensemble (NPT) equilibrium for 100000 steps, respectively, with the couplingconstant of 0.1 ps and the duration of 100ps. Finally, the free moleculardynamics simulation was performed. The process consisted of 50000000 steps, thestep length was 2fs, and the total duration was 100ns. After the simulation wascompleted, the built-in tool of the software was used to analyze thetrajectory, and the root-mean-square variance (RMSD), root-mean-squarefluctuation (RMSF) and protein rotation radius of each amino acid trajectorywere calculated, combined with the free energy topography and other data.

S2 Supplementary Text

## S2.1 LA-LYTAC^Biotin^ mediates the internalization and degradation of NA650


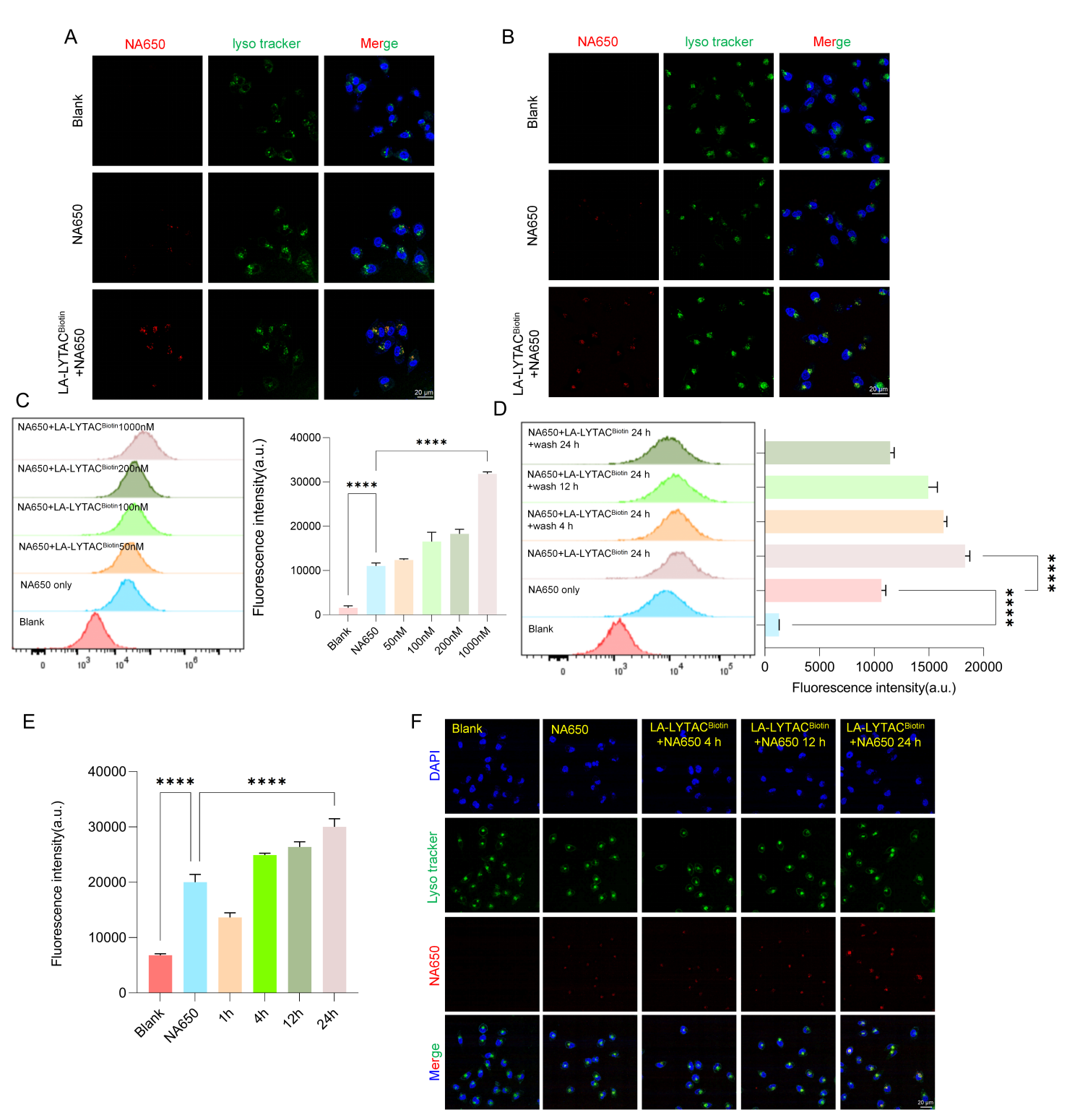


**Figure S1.** LA-LYTAC^Biotin^ mediates the internalization and degradation of NA650. **(A)** CLSM images of MDA-MB-231 cells incubated with 50 nM NA650 and LA-LYTAC^Biotin^ at 37°C for 24 h. Red: NA650; Green: LysoTracker; Blue: Hoechst; Illustration: DIC; Merge: Superimposition of different fluorescence channels. Scale bar = 20 μm. **(B)** CLSM images of HeLa cells incubated with 50 nM NA650 and LA-LYTACBiotin at 37°C for 24 h. **(C)** Flow cytometry analysis of NA650 internalization in MDA-MB-231 cells treated with 50 nM NA650 and different concentrations of LA-LYTAC^Biotin^ (50, 100, 200, 1000 nM) for 24 h. (n = 3 per group). **(D)** Flow cytometry analysis of NA650 uptake in MDA-MB-231 cells assisted by LA-LYTAC^Biotin^. Cells were treated with 1000 nM LA-LYTACBiotin and 50 nM NA650 for 24 h and then cultured in fresh medium for the specified time points (24, 12, 4 h). (n = 3 per group). **(E)** HeLa cells were treated with 50 nM NA650 and 1000 nM LA-LYTAC^Biotin^ for varying durations (24, 12, 4, and 1 hour), followed by flow cytometry analysis to assess NA650 internalization. (n = 3 per group). **(F)** CLSM images of HeLa cells incubated with 50 nM NA650 and LA-LYTAC^Biotin^ at 37°C for different times (4, 12, 24 h).

## S2.2 LA-LYTAC^Ab^ promotes PD-L1 degradation in MDA-MB-231 and 4T1 cell lines


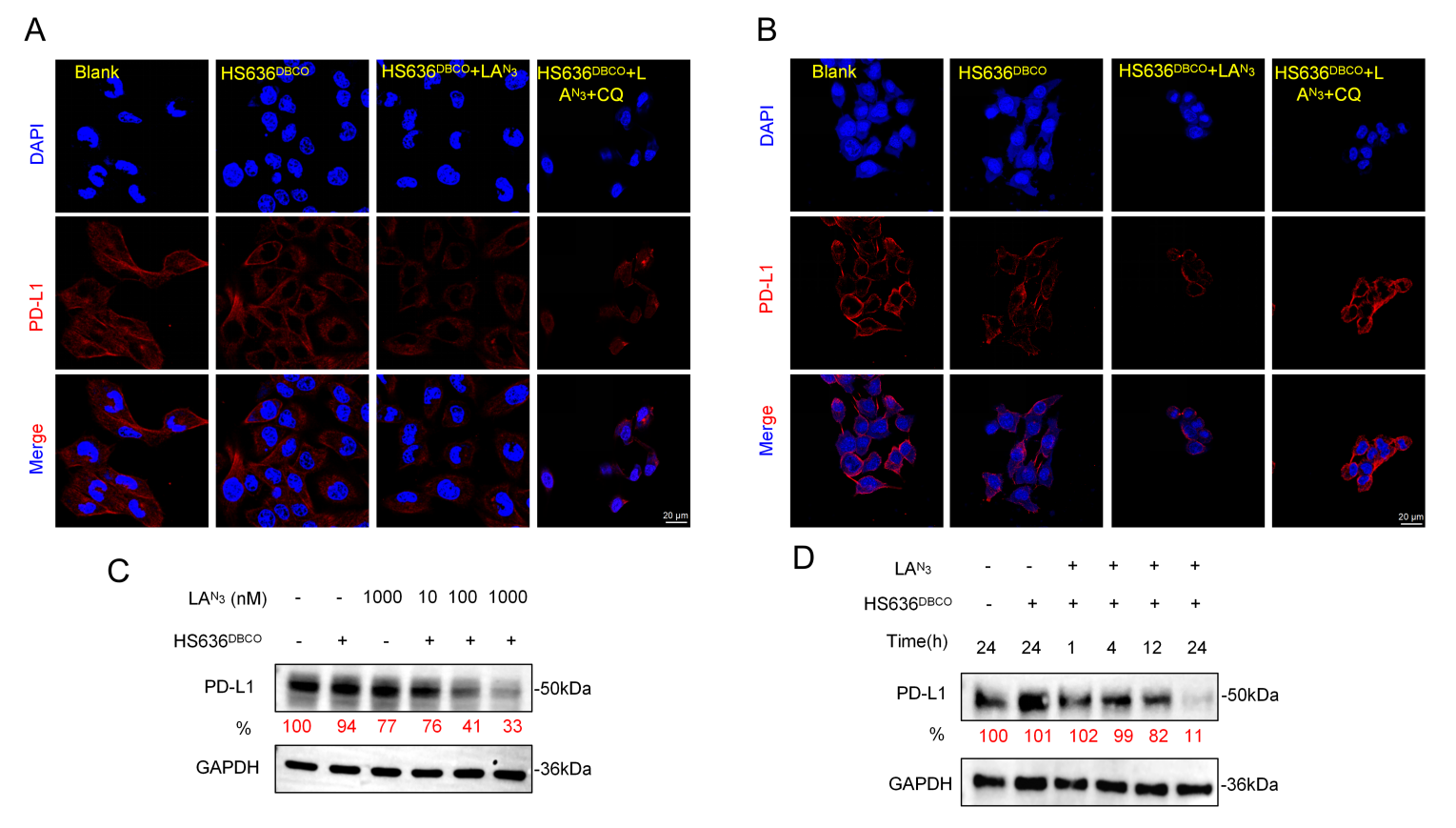


**Figure S2.** LA-LYTAC^Ab^ promotes PD-L1 degradation in MDA-MB-231 and 4T1 cell lines. **(A)** Confocal microscopy images of PD-L1 degradation in MDA-MB-231 cells after treatment with 10 nM HS636^DBCO^ and 1000 nM LA^N3^ for 24 hours. **(B)** Confocal microscopy images of PD-L1 degradation in 4T1 cells after treatment with 10 nM HS636^DBCO^ and 1000 nM LA^N3^ for 24 hours. **(C)** Western blot analysis of total PD-L1 levels in 4T1 cells treated with 10 nM HS636^DBCO^ and 10 nM, 100 nM, or 1000 nM LA^N3^ for 24 h. **(D)** Time course of cell surface PD-L1 degradation in MDA-MB-231 cells incubated with 10 nM HS636^DBCO^ and 1000 nM LA^N3^ for 1, 4, 12, and 24 hours.


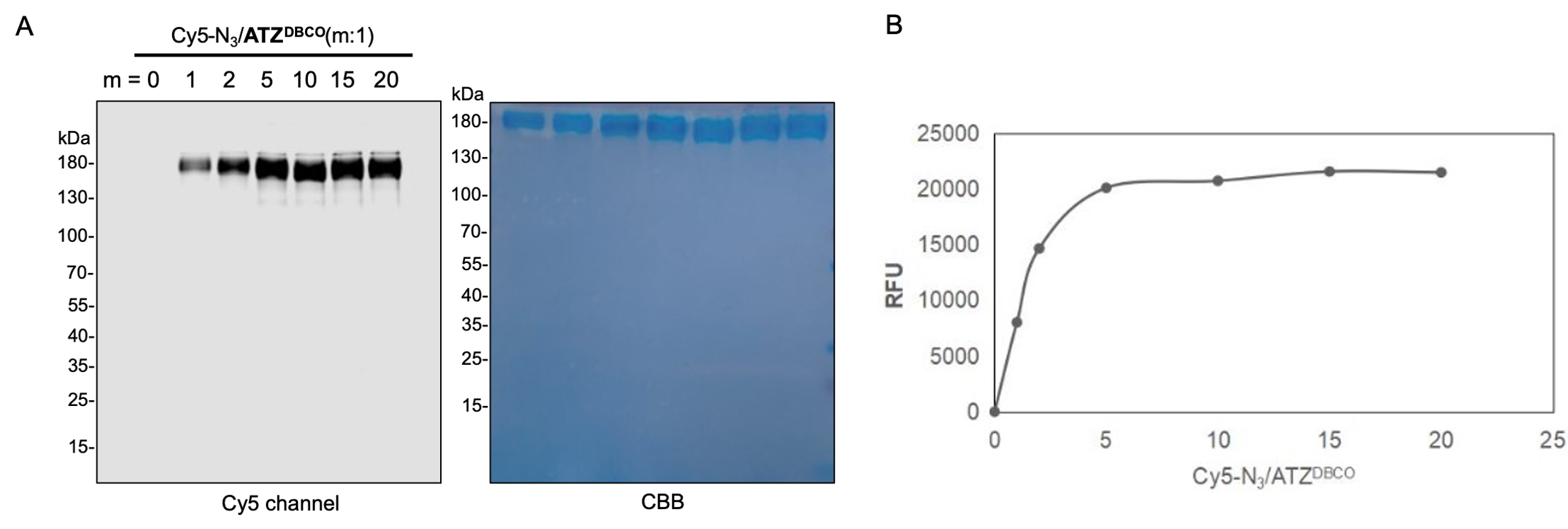


**Figure S3**. (A) **ATZ^DBCO^** was clicked with Cy5-N3 instead of **LAT1^N3^** for 2 days at room temperature, separated by SDS-PAGE and visualized by in-gel fluorescence scanning (FL) as well as Coomassie brilliant blue staining (CBB). Optimization of Cy5-N3 concentrations for full conversion of DBCO on **ATZ^DBCO^**. (B) The labeling ratio of Cy5-N3 (indicating LAT1 labeling ratio) on ATZ was estimated by determining the Dye : Protein molar ratio. The resulting ATZ : Cy5 ratio was calculated to be 5 under current labeling conditions.

## S2.3 LA-LYTAC^Ab^ promotes the degradation of EGFR in HeLa and 4T1 cell lines


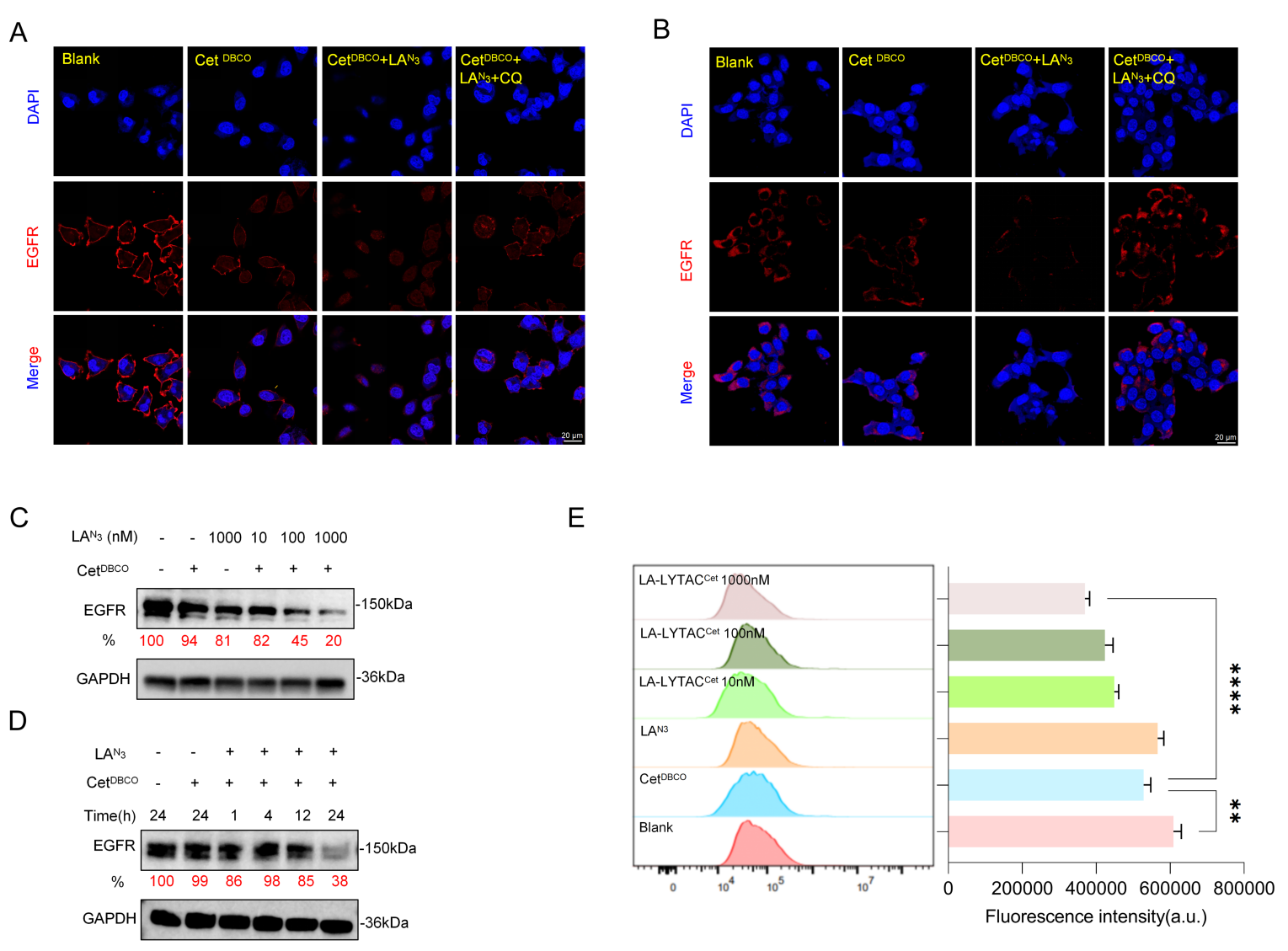


**Figure S4.** LA-LYTAC^Ab^ promotes the degradation of EGFR in HeLa and 4T1 cell lines. **(A)** Confocal microscopy images of EGFR degradation in HeLa cells after treatment with 10 nM Cet^DBCO^ and 1000 nM LA^N3^ for 24 hours. **(B)** Confocal microscopy images of EGFR degradation in 4T1 cells after treatment with 10 nM Cet^DBCO^ and 1000 nM LA^N3^ for 24 hours. **(C)** Western blot analysis of total EGFR levels in 4T1 cells after treatment with 10 nM Cet^DBCO^ and 10 nM, 100 nM or 1000 nM LA^N3^ for 24 hours. **(D)** Time course of surface EGFR degradation in 4T1 cells after incubation with 10 nM Cet^DBCO^ and 1000 nM LA^N3^ for 1, 4, 12 and 24 hours. (E) Flow cytometry analysis of EGFR degradation in 4T1 cells after treatment with different concentrations of LA-LYTAC^Cet^, LA^N3^ and Cet^DBCO^. (n = 3 per group).


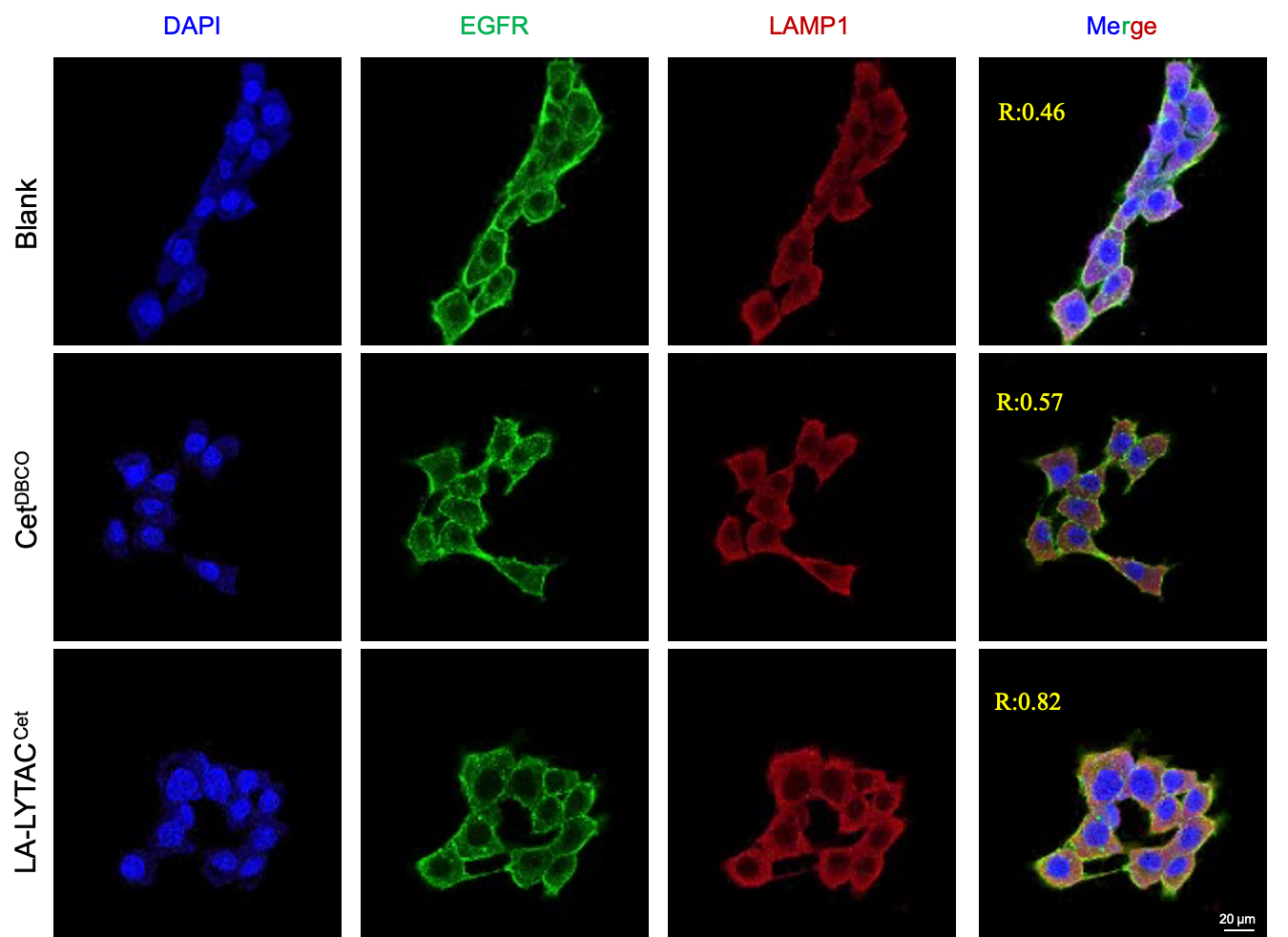


**Figure S5.** Confocal microscopy images showing lysosomal co-localization of EGFR in HeLa cells after treatment with 10 nM Cet^DBCO^ and 1000 nM LA^N3^ for 24 h. Cells were immunostained for EGFR and the lysosomal marker LAMP1, and co-localization between EGFR and LAMP1 was quantified using Pearson’s correlation coefficient. Scale bar: 20 µm.

## S2.4 LA-LYTAC^Ab^ promotes protein degradation in a LAT1 (SLC7A5)-dependent manner


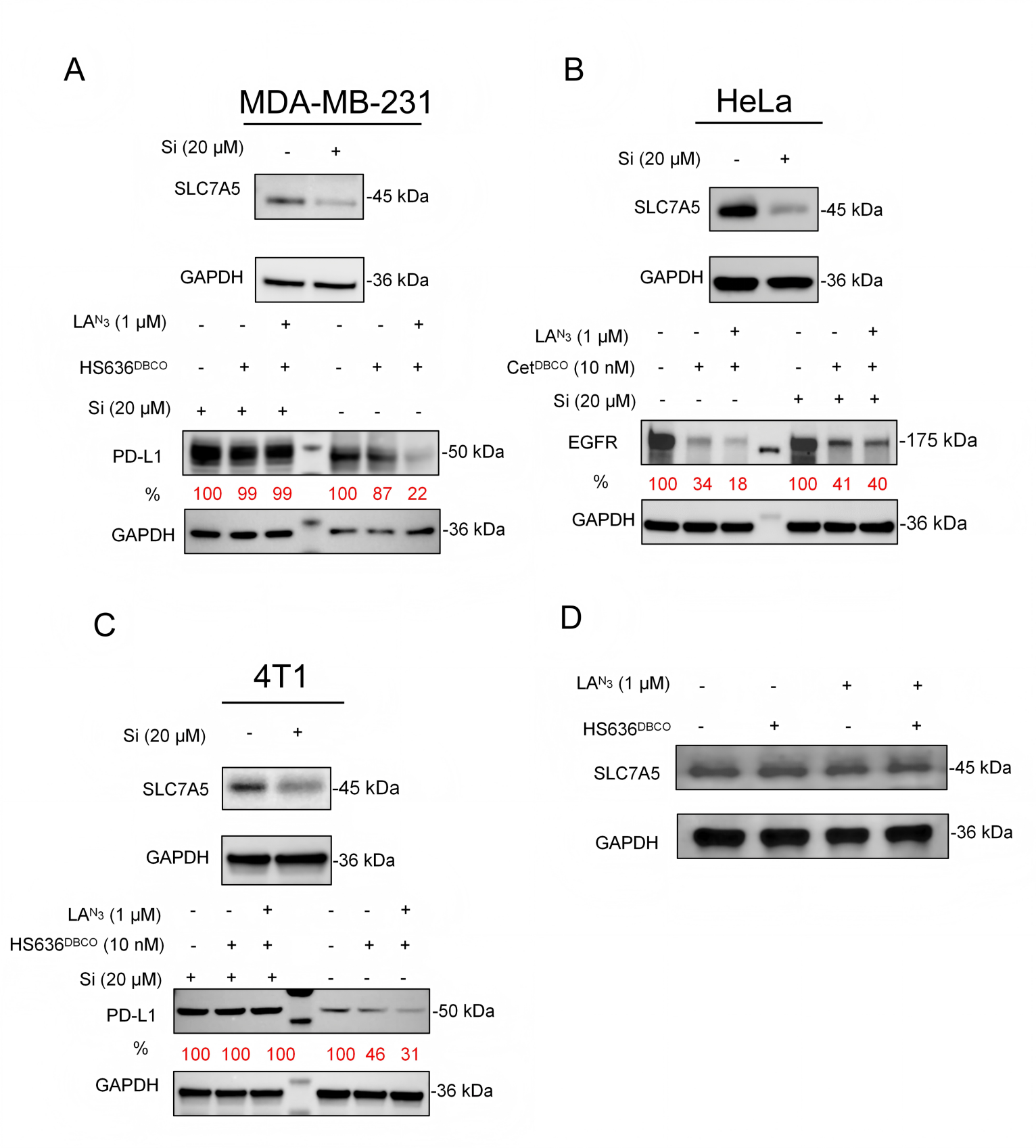


**Figure S6.** LA-LYTAC^Ab^ promotes protein degradation in a LAT1 (SLC7A5)-dependent manner. **(A)** Western blot analysis of PD-L1 expression levels in MDA-MB-231 cells with SLC7A5 knockdown and various drug treatments. **(B)** Western blot analysis of EGFR expression levels in HeLa cells with SLC7A5 knockdown and various drug treatments. **(C)** Western blot analysis of PD-L1 expression levels in 4T1 cells with SLC7A5 knockdown and various drug treatments. **(D)** Western blot analysis of SLC7A5 protein expression levels upon treatment with different drugs.

## S2.5 Confocal microscopy observation of integrin alpha 5 degradation in 4T1 cells


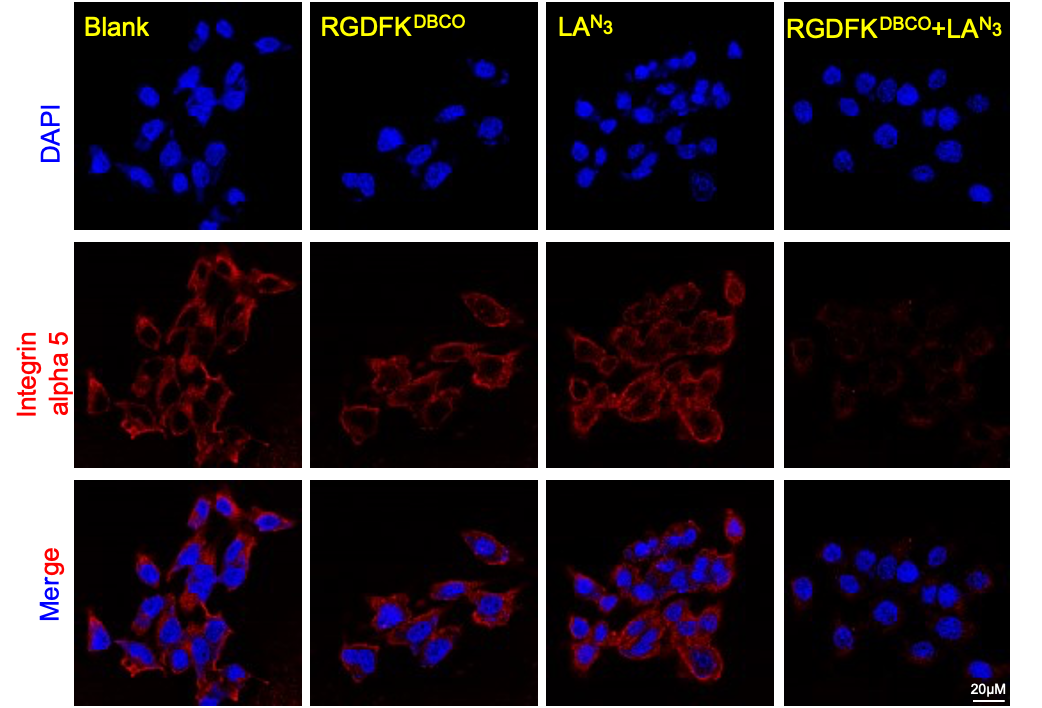


**Figure S7.** Confocal microscopy observation of integrin alpha 5 degradation in 4T1 cells treated with 1000 nM RGDFK^DBCO^ and 1000 nM LAN3 for 24 hours. Red: Integrin beta 3; Blue: Hoechst; Illustration: DIC; Merge: Superimposition of different fluorescence channels. Scale bar = 20 μm.


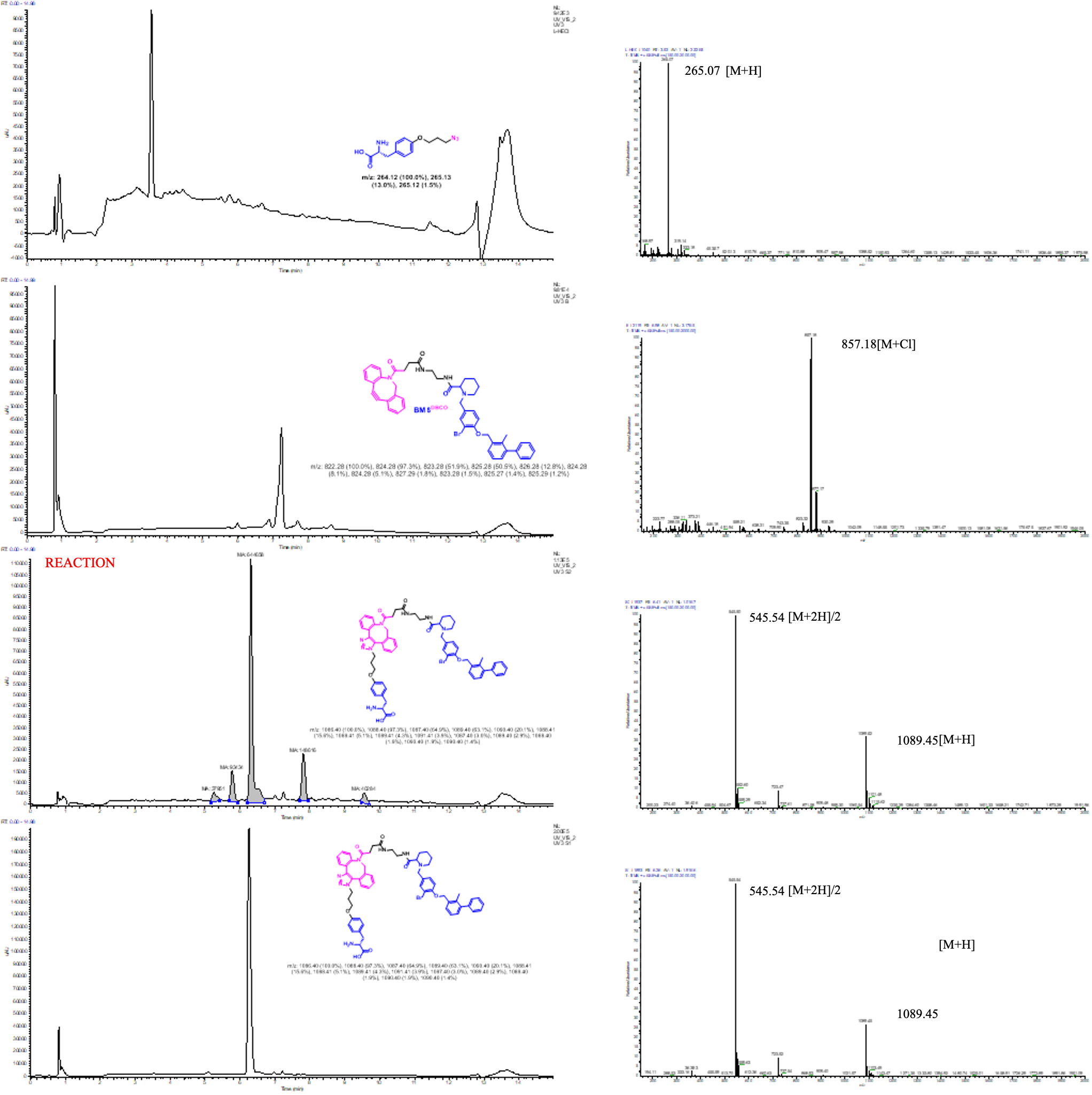


**Figure S8. LC–MS comparison of the crude click reaction mixture and purified product.**
The crude mixture after the click reaction and the purified product were analyzed by LC–MS. The main chromatographic peak and corresponding mass signal of the crude reaction mixture were largely consistent with those of the purified product, suggesting that purification did not markedly change the major product composition. This result supports the efficient formation of the desired click-conjugated product.


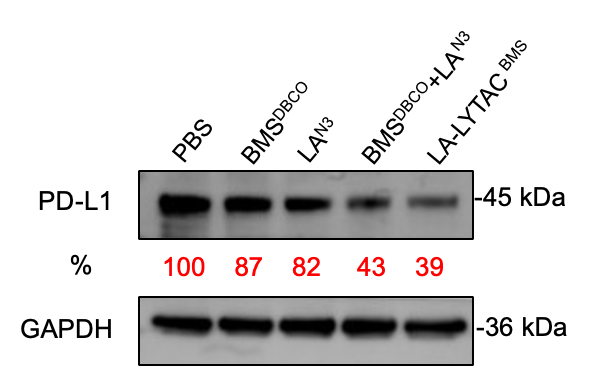


**Figure S9. Activity comparison between crude and purified click-conjugated products.**

Western blot analysis of target protein degradation after treatment with vehicle, crude click reaction mixture, or purified product at the indicated concentrations. Target protein levels were normalized to the loading control, and relative intensities are shown below each lane. The crude and purified products exhibited comparable degradation activity, supporting that the click reaction efficiently generated the active conjugated product.

## S2.6 Representative whole and magnified views of TUNEL-stained tumor


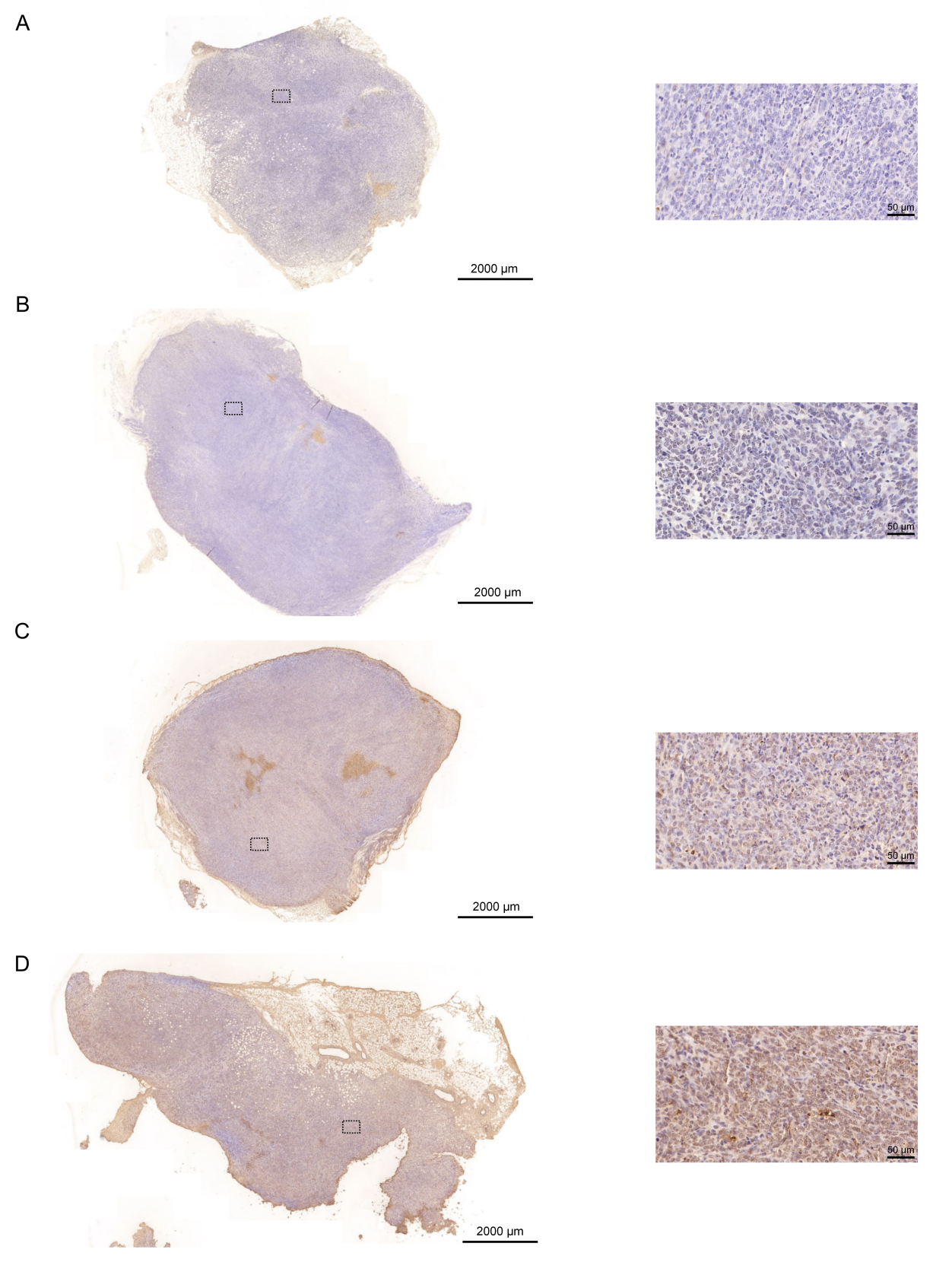


**Figure S10.** Representative whole and magnified views of TUNEL-stained tumor sections from mice in different groups. **(A)** Whole and magnified views of TUNEL-stained sections from mice treated with PBS. **(B)** Whole and magnified views of TUNEL-stained sections from mice treated with LA^N3^. **(C)** Whole and magnified views of TUNEL-stained sections from mice treated with Atezolizumab. **(D)** Whole and magnified views of TUNEL-stained sections from mice treated with LA-LYTAC^ATZ^.

## S2.7 Immunofluorescence analysis of cell surface PD-L1 protein


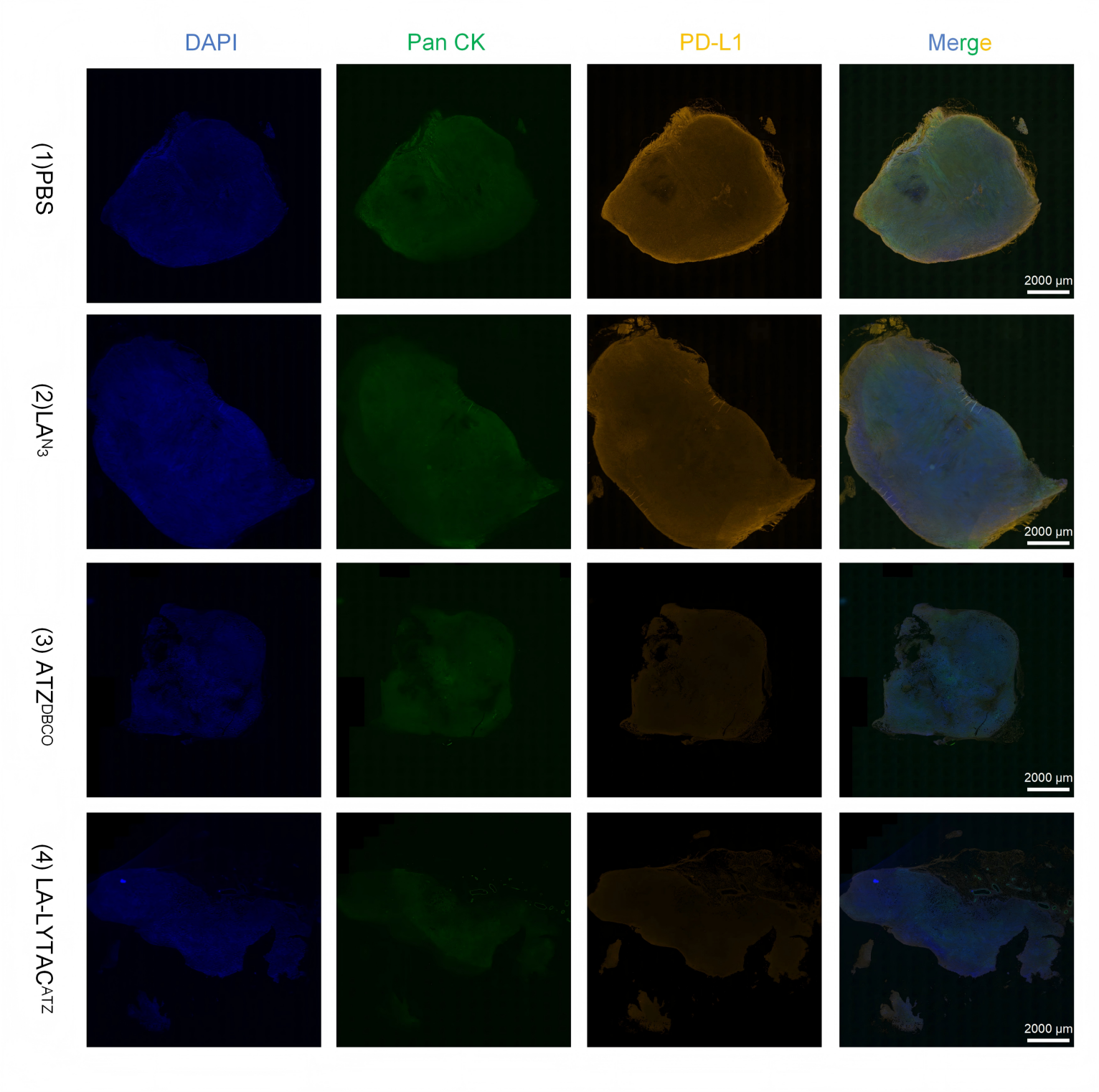


**Figure S11.** Immunofluorescence analysis of cell surface PD-L1 protein upon different drug treatments. DAPI (blue), Pan-CK (green), PD-L1 (yellow). (Scale bar: 2000 μm).

## S2.8 Mass spectrum of RGDFK^DBCO^


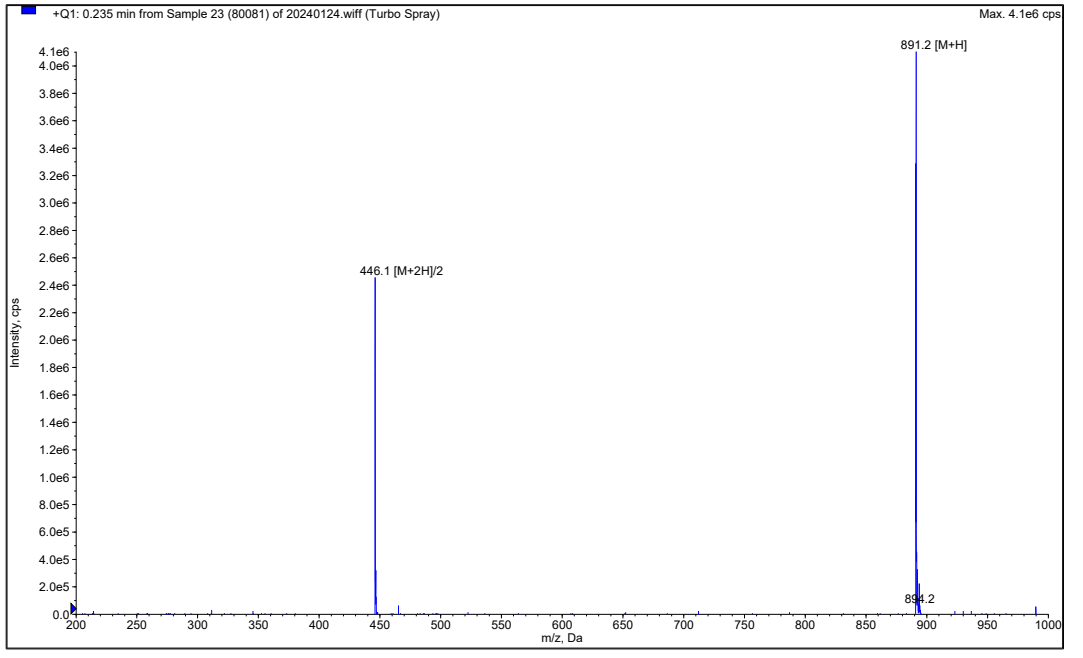


**Figure S12.** Mass spectrum of RGDFK^DBCO^.

## S2.9 Mass spectrum of BMS^DBCO^


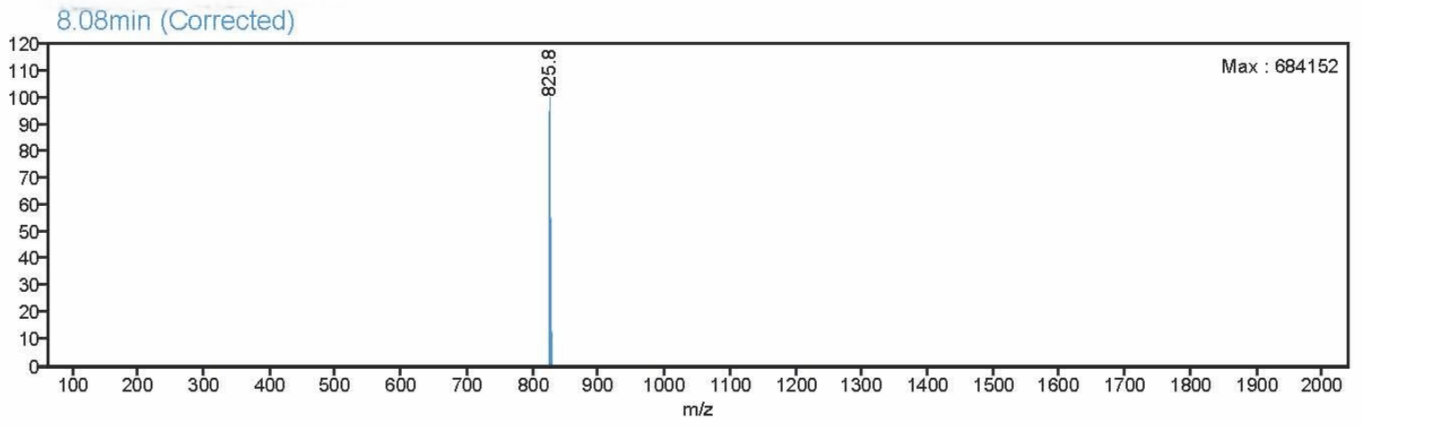


**Figure S13.** Mass spectrum of BMS^DBCO^.

## S2.10 Mass spectrum of LA^Biotin^


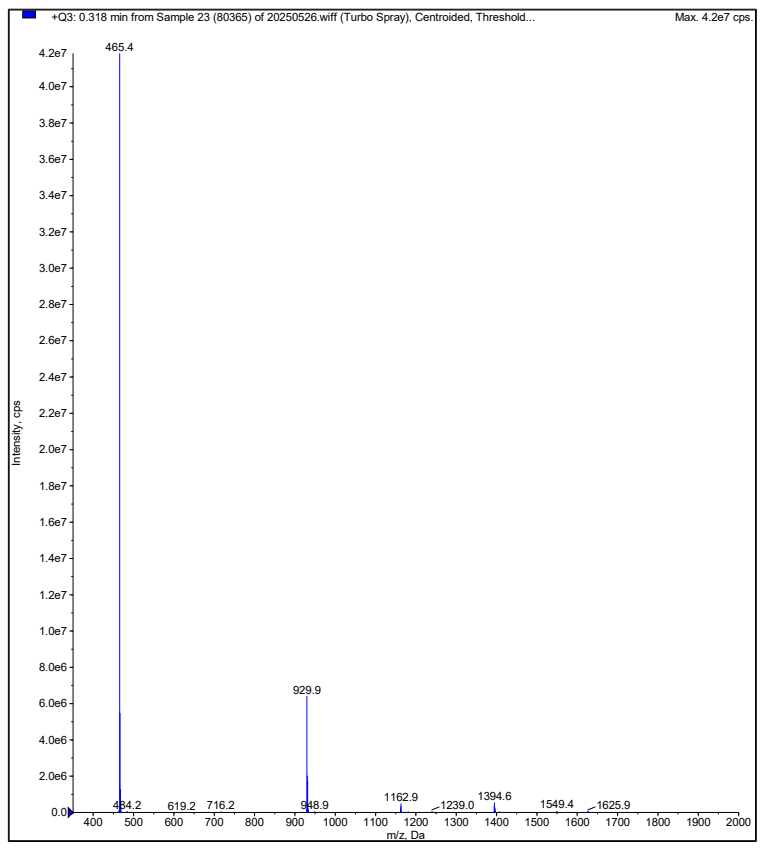


**Figure S14.** Mass spectrum of LA^Biotin^.

## S2.11 NMR and Mass Spectrum of LA^N3^


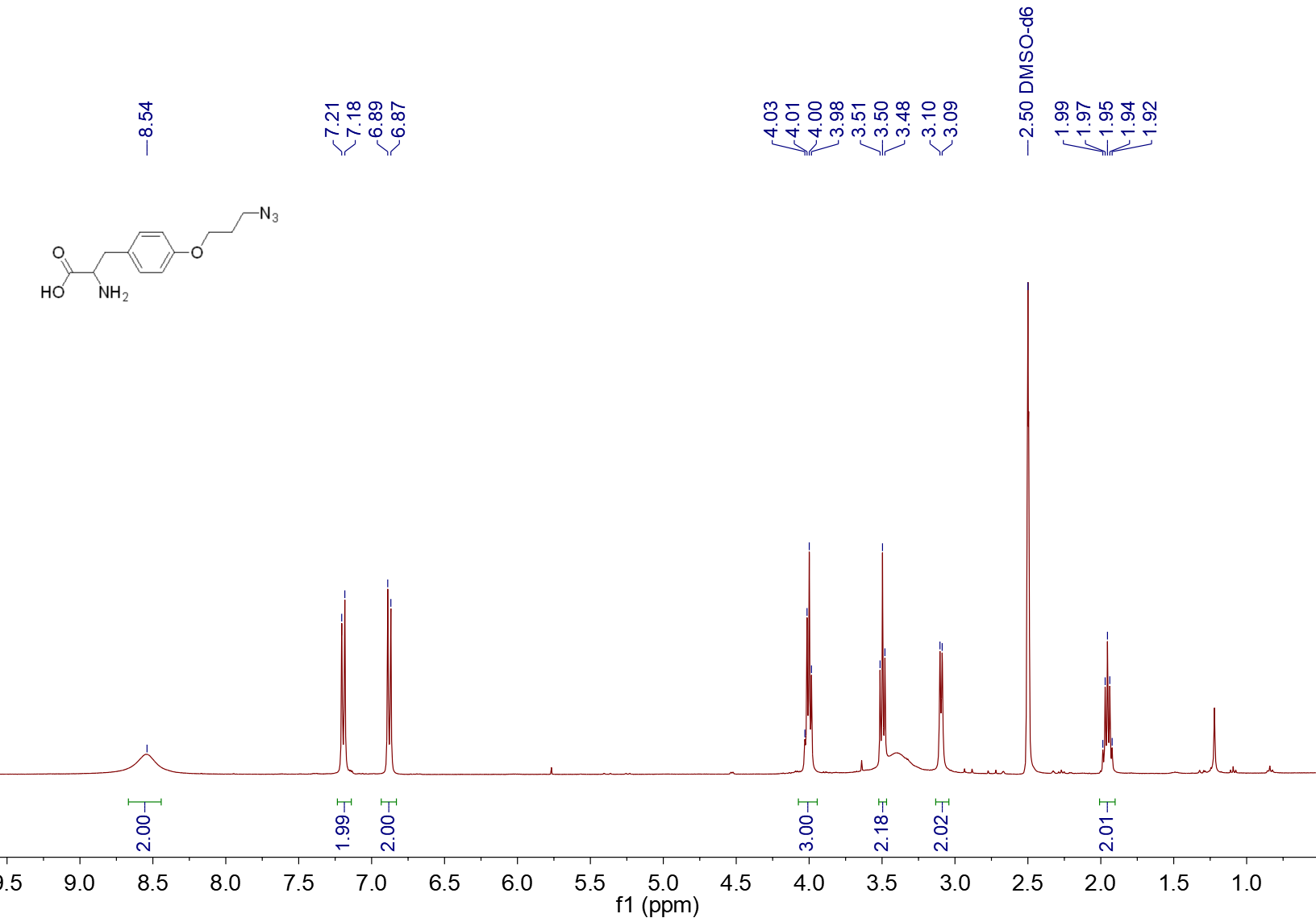


**Figure S15.** ^1^H NMR spectrum of LA^N3^.


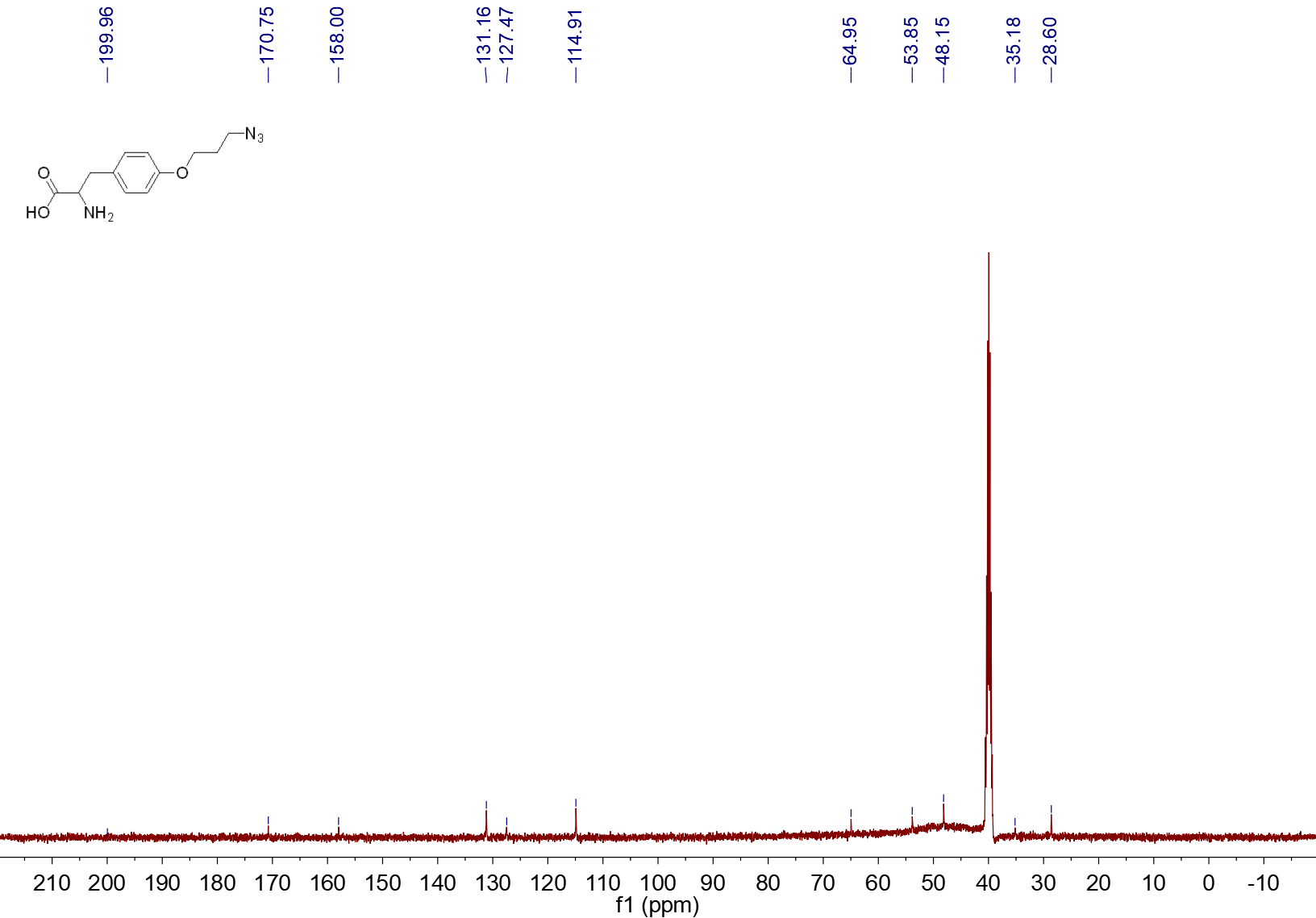


**Figure S16.** ^13^C NMR spectrum of LA^N3^.

**Figure S17.** Mass spectrum of LA^N3^.

# S3 Tables

**Supplementary Table 1. Antibodies used for western blotting.**

| Reagent | Source | Identifier | Batch | Dilution |
| --- | --- | --- | --- | --- |
| PD-L1 | HUABIO | JJ08-95 | H681262061 | 1:5000 |
| Integrin alpha 5 | HUABIO | JJ08-94 | H650105005 | 1:2000 |
| Integrin beta 3 | HUABIO | PSH09-27 | H650607111 | 1:2000 |
| LAT1(SLC7A5) | HUABIO | PSH09-28 | HA723086 | 1:2000 |
| EGFR | HUABIO | B6-E5-D9 | HK1107 | 1:5000 |
| Phospho-EGFR (Y1092) | HUABIO | SJ0194 | H680663023 | 1:10000 |
| AKT1/2/3 | HUABIO | JE75-09 | H651468036 | 1:2000 |
| Phospho-AKT (S473) | HUABIO | SY28-05 | H651506050 | 1:5000 |
| GAPDH | HUABIO | SA30-01 | H650617029 | 1:50000 |
| ERK1/2 | Proteintech | 16443-1-AP | 00018061 | 1:2000 |
| Phospho-ERK1/2 (Thr202/Tyr204) | Affinity | #AF1015 | #5493f17 | 1:1000 |
| HRP Conjugated Goat anti-Mouse IgG | HUABIO | HA1006 | H650100002 | 1:50000 |
| HRP Conjugated Goat anti-Rabbit IgG | HUABIO | HA1001 | H651762016 | 1:50000 |

**Supplementary Table 2. Antibodies used for immunofluorescence**

| Reagent | Source | Identifier | Batch | Dilution |
| --- | --- | --- | --- | --- |
| PD-L1 | HUABIO | JJ08-95 | H681262061 | 1:200 |
| Integrin alpha 5 | HUABIO | JJ08-94 | H650105005 | 1:200 |
| Integrin beta 3 | HUABIO | PSH09-27 | H650607111 | 1:200 |
| EGFR | HUABIO | B6-E5-D9 | HK1107 | 1:400 |
| 488-Goat Anti-Rabbit | Proteintech | RGAR002 | 20001066 | 1:500 |
| 647-Goat Anti-Rabbit | Proteintech | RGAR005 | 20001140 | 1:500 |
| 647-Goat Anti-Mouse | Proteintech | RGAM005 | 20001124 | 1:500 |

**Supplementary Table 3. Antibodies used for flow cytometry**

| Reagent | Source | Identifier |
| --- | --- | --- |
| PE anti-mouse CD8a Antibody | Biolegend | 100707 |
| APC anti-mouse CD4 Antibody | Biolegend | 100515 |
| Zombie Aqua™ Fixable Viability Kit | Biolegend | 423101 |
| Mouse FcR Blocking,Reagent | STARTER | S0B0599 |

**Supplementary Table 4. Antibodies used for IHC**

| Reagent | Source | Identifier | Batch | Dilution |
| --- | --- | --- | --- | --- |
| Anti-Ki67 Antibody | HUABIO | SR00-02 | H680619015 | 1:5000 |
| Tunel reagent kit | RecordBio | RC-012 |  |  |
| HRP Conjugated Goat anti-Rabbit IgG | HUABIO | HA1001 | H651762016 | 1:200 |
